# ﻿A new ant species of the genus *Carebara* Westwood, 1840 (Hymenoptera, Formicidae, Myrmicinae) with a key to Chinese species

**DOI:** 10.3897/zookeys.1190.110552

**Published:** 2024-01-22

**Authors:** Zhi-yu Liu, Ying Zhong, Yu-yuan Huang, Hao Ran, Fan Song

**Affiliations:** 1 Department of Entomology and MOA Key Lab of Pest Monitoring and Green Management, College of Plant Protection, China Agricultural University, Beijing 100193, China China Agricultural University Beijing China; 2 Shenzhen Jianwen Foreign Language School, Longgang, Shenzhen, Guangdong 518116, China Shenzhen Jianwen Foreign Language School Shenzhen China; 3 Wuchuan No.1 Middle School, Wuchuan, Guangdong 524500, China Wuchuan No.1 Middle School Wuchuan China; 4 Key Laboratory of Ecology of Rare and Endangered Species and Environmental Protection (Guangxi Normal University), Ministry of Education, Guilin 541004, China Guangxi Normal University Guilin China

**Keywords:** *Carebaralaeviceps*, China, East Asia, new species, Sichuan Province, taxonomy

## Abstract

A new Chinese ant species *Carebaralaeviceps***sp. nov.** is described based on the major and minor workers. This species is most similar to *C.lusciosa* (Wheeler, 1928) due to a spineless propodeum, the absence of horns, and a smooth head capsule. It is distinguished by the following features: (1) antenna 10-segmented; (2) katepisternum rugose-reticulate; (3) in major workers, lateral sides of head in full-face view parallel; (4) metanotal groove distinct, anterodorsal corner forming an acute tooth behind metanotal groove. Moreover, an updated key to Chinese *Carebara* species is presented based on major workers, with a checklist comprising a total of 36 Chinese *Carebara* species and subspecies. Morphological structures and scanning electron micrographs of the newly discovered species’ minor and major workers are provided.

## ﻿Introduction

The genus *Carebara* Westwood, 1840 is a large genus of ants that contains 234 valid species (including 9 fossil species) and 22 subspecies (Bolton 2023). It is a worldwide genus mainly recorded in the tropical and subtropical regions (Bharti and Kumar 2013; Azorsa and Fisher 2018). They nest in soil or termite mounds, and some species also inhabit rotten wood (Bharti and Kumar 2013). Most members of *Carebara* are minute in size and subterranean, mainly feeding on dead insects and other invertebrates. Some species are aggressive ground predators with mass raiding habits (Moffett 1988), like those of the former genus *Pheidologeton* Mayr, 1862. Studies regarding the ethology and life cycle of *Carebara* are still limited.

The genus was established based on the type species *C.lignata* Westwood, 1840. It was originally incorporated into the formerly valid subfamily Attidae after its establishment (Smith 1858). Later, the taxonomic status underwent several changes among the tribes Solenopsidini (Forel 1893; Emery 1895; Wheeler 1910, 1922; Kusnezov 1964) Pheidologetini (Emery 1913, 1914, 1924; Bolton 1994), and the *Pheidologeton* genus group (Ettershank 1966; Bolton 1987) due to the incomplete records for different castes and polymorphism. The genus *Carebara* was considered as a senior synonym of the genera *Aeromyrma* Forel, *Afroxyidris* Belshaw & Bolton, *Amauromyrmex* Wheeler, *Aneleus* Emery, *Crateropsis* Patrizi, *Erebomyrma* Wheeler, *Hendecatella* Wheeler, *Idrisella* Santschi, *Lecanomyrma* Forel, *Neoblepharidatta* Sheela & Narendran, *Nimbamyrma* Bernard, *Oligomyrmex* Mayr, *Paedalgus* Forel, *Parvimyrma* Eguchi & Bui, *Pheidologeton* Mayr, *Solenops* Karavaiev, *Spelaeomyrmex* Wheeler, and *Sporocleptes* Arnold (Fischer et al. 2014), and finally placed in Crematogastrini by Ward et al. (2015) based on a comprehensive phylogenetic analysis of 11 genes.

Taxonomic changes at the genus level have superseded some early regional revisions of *Carebara* species. Moreover, the lack of a comprehensive revision, especially for Old World species, has created difficulties in species identification (Fischer et al. 2015). To address these issues, recent regional studies have been conducted in India (Bharti and Kumar 2013; Bharti et al. 2014; Akbar and Bharti 2017), Saudi Arabia (Sharaf and Aldawood 2013), the Afrotropical region (Fischer et al. 2014, 2015), Brazil (Baccaro et al. 2015), Madagascar (Azorsa and Fisher 2018), and Australia (Heterick 2021). The studies about species groups initially concentrated on New World species, and Fernández (2004, 2010) reviewed American species and proposed five species groups based on the morphology of worker caste. In the Old World, the species groups of *Carebara* were first studied by Bharti and Kumar (2013). They placed 11 Indian species into three groups and recommended the fusion of the *C.concinna* group with the *lignata* group based on the eyeless minor worker of *C.asina* (Forel, 1902). Fischer et al. (2014) established the *C.polita* group, which shares morphological similarities with *Pheidologeton*, and included six Afrotropical species and two Neotropical species. This study also synonymized *Pheidologeton* with *Carebara*. Later, Fischer et al. (2015) established the *phragmotica* group, and the *acutispina* group was proposed by Hosoishi et al. (2022), both including phragmotic species.

In China, *C.castanea* Smith, 1858 was the first Chinese *Carebara* species to be described from Hong Kong. Subsequently, *C.sauteri* (Forel, 1912) and *C.yanoi* (Forel, 1912), followed by the queen caste-established species *C.amia* (Forel, 1913), were all collected in Taiwan. Wheeler (1921) described a species from Zhejiang, namely *C.vespillo* (Wheeler, 1921). Later, Wheeler (1928) described three new species and two new subspecies of China; among them, *Oligomyrmexsilvestriitaiponicus* was raised to species status by Bolton (1995) and is now known under the name *C.taiponica* (Wheeler, 1928). Another species *Oligomyrmexsilvestrii* was considered a secondary homonym of *Aneleussilvestrii* Santschi, 1914, later renamed *Oligomyrmexwheeleri* Ettershank, 1966, but now known as *C.wheeleri* (Ettershank, 1966). The remaining three taxa are *C.lusciosa*, *C.polyphemus* (Wheeler, 1928), and *C.capreolalaeviceps* (Wheeler, 1928).

Later, Wu and Wang (1995) revised some ant genera from the Chinese mainland, including the former valid genera *Pheidologeton* and *Oligomyrmex* (now both in *Carebara*). They also described three new species, namely *C.hunanensis* (Wu & Wang, 1995), *C.jiangxiensis* (Wu & Wang, 1995), and *C.pseudolusciosa* (Wu & Wang, 1995). It is worth mentioning that Wu and Wang (1995) erroneously illustrated these three species in relation to eye position, tooth numbers, and cephalic indices (Xu 2003). Zhou and Zheng (1997) as well as Li and Tang (1986) conducted comprehensive studies on *Carebara* from Guangxi Province, and described four new species, *C.nanningensis* (Li & Tang, 1986), *C.latinoda* (Zhou & Zheng, 1997), *C.melasolena* (Zhou & Zheng, 1997), and *C.trechideros* (Zhou & Zheng, 1997).

The very first comprehensive revision of former *Oligomyrmex* species in China was presented by Xu (2003), who studied this genus in more depth, conducting a revision of 26 species in China, and described eight new species, namely *C.altinodus* (Xu, 2003), *C.curvispina* (Xu, 2003), *C.striata* (Xu, 2003), *C.acutispina* (Xu, 2003), *C.obtusidenta* (Xu, 2003), *C.bihornata* (Xu, 2003), *C.rectidorsa* (Xu, 2003) and *C.reticapita* (Xu, 2003) from China. Additionally, Xu excluded *C.cribriceps* (Wheeler, 1927). Despite Zhou’s (2001) description of *C.cribriceps* in Guangxi, Zhou’s illustration of this species displays a minor concavity in the posterior margin of the head, in contrast to Wheeler’s (1927) account, which distinctly portrays a pronounced concavity in the posterior margin. This disparity suggests that the species documented by Zhou may be an undescribed species from China (Xu 2003). Later, a new and different species, *C.zengchengensis* (Zhou et al., 2006) was described from Guangdong.

*Carebara* species from Taiwan were mostly studied by Terayama (2009), who made several revisions and described two new species *C.qianliyan* Terayama, 2009 and *C.sakamotoi* Terayama et al., 2012. Currently, there are a total of 36 valid species and subspecies in China.

As a contribution to the taxonomy of the *Carebara* species of China, we report a new species: *C.laeviceps* sp. nov. High-resolution images and scanning electron micrographs (SEM) of the minor and major workers of the new species are provided. An updated key to Chinese *Carebara* species is also provided based on the major worker.

## ﻿Material and methods

All samples were collected from Kaijiang County, Sichuan Province, China by direct sampling on the ground and preserved in 75% EtOH, then deposited in the Forest Insect Herbarium, Ant Specimen Branch of Southwest Forestry University, Kunming, China (**SWFU**). Specimens were observed under a Phenix XSP-02 microscope. Photographs were taken by Samsung SM-N9860, and SEM photographs were taken by a FEI Quanta 450 at 12.50 kV. To observe the microstructure and preserve the specimens, some of the specimens were disassembled before observation under SEM. The specimens were sputter-coated with gold for 30 min. Image stacking using Helicon Focus software. Morphological terminology and standard measurements mostly follow Bolton (1994), all measurements are given in millimeters:

**HL** Head Length. Maximum length from the mid-point of the anterior clypeal margin to the mid-point of the posterior margin measured in full-face view.

**HW** Head Width. Maximum width of the head measured in full-face view.

**EL** Eye Length. Maximum length of the eye measured in lateral view.

**SL** Scape Length. Maximum length of the antennal scape measured in full-face view.

**WL** Weber’s Length. Maximum diagonal length from the most anterior point of the pronotal slope to the most posteroventral margin of propodeal lobe measured in lateral view.

**PNW** Pronotum Width. Maximum width of pronotum measured in dorsal view.

**PNH** Pronotum Height. Maximum height of pronotum measured in lateral view from index of procoxa to the highest point of the dorsal pronotum.

**MNH** Promesonotum Height. Maximum height of promesonotum measured in lateral view from the index of mesocoxa to the highest point of the dorsal pronotum.

**PDH** Propodeum Height. Maximum height of propodeum, measured in lateral view from the highest point of the dorsopropodeum perpendicular to a line that marks the lateroventral borders of the katepisternum and the propodeum.

**PTL** Petiolar Length. Maximum length of petiole measured in lateral view from most anteroventral point of the peduncle, at or below the propodeal lobe, to most posterodorsal point at the junction with helcial tergite.

**PTH** Petiolar Height. Maximum height of petiole measured in lateral view from the highest (median) point of the node, orthogonally to the ventral outline of the node.

**PTW** Petiolar Width. Maximum width of petiole measured in dorsal view.

**PPL** Postpetiolar Length. Maximum length of postpetiole measured in dorsal view from the anterior end of the node to the posterior end of the node.

**PPH** Postpetiolar Height. Maximum height of postpetiole measured in lateral view from the highest point of the node to the lowest point of the ventral process, often in an oblique line.

**PPW** Postpetiolar Width. Maximum width of postpetiole measured in dorsal view.

### ﻿Ratios

**CI** Cephalic index: HW / HL × 100;

**SI** Scape index: SL / HW × 100;

**EI** Eye index: EL / HW × 100;

**LPpI** Lateral postpetiole index: PPL / PPH × 100;

**DPpI** Dorsal postpetiole index: PPW / PPL × 100;

**PpWI** Postpetiole width index: PPW / PTW × 100;

**PpLI** Postpetiole length index: PPL / PTL ×100;

**PpHI** Postpetiole height index: PPH / PTH × 100;

**PPI** Postpetiole index: PPW / PNW × 100.

## ﻿Taxonomy

### ﻿Genus Carebara

Taxon classificationAnimaliaHymenopteraFormicidae

Westwood, 1840

DE64F4D9-152C-56DF-A0A8-5E3EC5F15C88

Carebara Westwood, 1840: 86. Type species: Carebaralignata Westwood, 1840: 86, Indonesia (Java). Indomalaya.
= Pheidologeton Mayr, 1862: 750. Synonymized by Fischer et al. 2014: 63.
= Oligomyrmex Mayr, 1867: 110. Synonymized by Fernández 2004: 194.
= Aeromyrma Forel, 1891: 198. Synonymized by Fernández 2004: 194.
= Aneleus Emery, 1900: 327. Synonymized by Fernández 2004: 194.
= Erebomyrma Wheeler, 1903: 138. Synonymized by Fernández 2004: 194.
= Paedalgus Forel, 1911: 217. Synonymized by Fernández 2004: 194.
= Lecanomyrma Forel, 1913: 56. Synonymized by Fernández 2004: 194.
= Spelaeomyrmex Wheeler, 1922: 9. Synonymized by Fernández 2004: 194.
= Hendecatella Wheeler, 1927: 93. Synonymized by Fernández 2004: 194.
= Amauromyrmex Wheeler, 1929: 1. Synonymized by Fischer et al. 2014: 63.
= Solenops Karavaiev, 1930: 207. Synonymized by Fernández 2004: 194.
= Idrisella Santschi, 1937: 372. Synonymized by Fischer et al. 2014: 66.
= Crateropsis Patrizi, 1948: 174. Synonymized by Fernández 2004: 194.
= Sporocleptes Arnold, 1948: 219. Synonymized by Fernández 2004: 194.
= Nimbamyrma Bernard, 1953: 240. Synonymized by Fernández 2004: 194.
= Afroxyidris Belshaw & Bolton, 1994: 631. Synonymized by Fernández 2004: 194.
= Neoblepharidatta Sheela & Narendran, 1997: 88. Synonymized by Fernández 2004: 194.
= Parvimyrma Eguchi & Bui, 2007: 40. Synonymized by Fernández 2010: 195.

#### Synopsis of members of Carebara from China.

**Figure 1.**
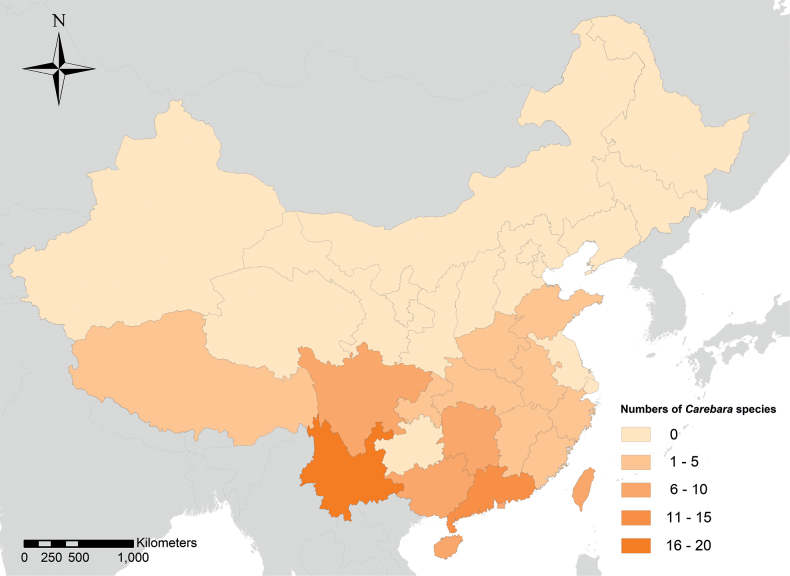
Map of the diversity of known Chinese *Carebara* species. Shades from pale to dark indicate species numbers from low to high.

Currently, there are 36 *Carebara* species and subspecies in China, with the majority in the southern and southwestern regions (Fig. 1). The highest diversity is observed in Guangdong, Guangxi, Yunnan, and Sichuan provinces (Xu 2003; Zhou and Zheng 1997).

Here we provide a brief overview of the provisional definition of Chinese *Carebara* species groups. In addition to the former *Pheidologeton* species, other *Carebara* members in China (except *C.amia*) align with the *concinna-lignata* group proposed by Bharti. It is worth mentioning that the criteria for classification vary among different studies. Bharti and Kumar (2013) suggested merging the *concinna* and *lignata* groups, while Fischer et al. (2015) retained the *lignata* group, defining its features based on Fernández (2004): workers typically small with 9-segmented antenna, mandibles 3- or 4-toothed, absent eyes and propodeal teeth, and a rounded dorsum of the propodeum. The queens are generally much larger than the workers. Hosoishi et al. (2022) established the *acutispina* group; however, it is highly probable that this is an artificial group within the *concinna-lignata* group. Due to the lack of comprehensive revisions of all castes (especially queens and males), the current definition of species groups relies predominantly on the morphology of the worker caste. However, this approach introduces uncertainties due to the potential influence of convergent evolution and a global investigation into *Carebara* species, utilizing molecular data, is imperative for a more accurate understanding of the phylogenetic relationship between groups. To prevent the proposal of multiple species groups, our definition is mainly based on Bharti and Kumar (2013).

There are 11 species and subspecies of China belonging to the previously valid genus *Pheidologeton*: *C.affinis* (Jerdon, 1851), *C.diversa* (Jerdon, 1851), *C.diversadraco* (Santschi, 1920), *C.diversalaotina* (Santschi, 1920), *C.latinoda*, *C.melasolena*, *C.nanningensis*, *C.trechideros*, *C.vespillo*, *C.yanoi*, *C.zengchengensis*. These species are identified by their 11-segmented antennae, distinct polymorphic worker castes, and multifaceted eyes (Fischer et al. 2015). Fischer et al. (2014) indicated that former *Pheidologeton* species would be split into two groups: one with a polymorphic worker caste and the other with a dimorphic worker caste, and the 11 Chinese taxa mentioned above belong to the former group.

The *lignata* group was originally established by Fernández (2004) to group New World species characterized by minor workers with 9-segmented antenna and the absence of eyes. The minor workers of this group mainly lack propodeal spines. Major workers present or absent. Members of the *concinna* group with dimorphic workers, eyes present in both major and minor workers, propodeum armed or only angulated, antenna 9- to 11- (rarely 8-) segmented. Bharti and Kumar (2013) suggested redefining the *concinna* group including the *lignata* group; this alteration was based on the observation that the major worker of *C.asina* aligned with the *concinna* group, whereas the minor worker lacked eyes and propodeal spines, consistent with the traditional *lignata* group proposed by Fernández (2010).

Certain Chinese *Carebara* species, like *C.bihornata* and *C.sakamotoi*, also form a bridge between the *lignata* and *concinna* groups. Similar to *C.asina*, *C.bihornata* exhibit eyeless minor workers with an unarmed propodeum, while the major workers accord with the features of *concinna* group. Some species provide additional insights into the *concinna-lignata* group, such as *C.capreola* and *C.curvispina*, both characterized by eyeless major and minor workers. This suggests that the features of the *concinna-lignata* group include: (1) workers monomorphic or dimorphic; (2) antenna 9- to 11- (rarely 8-) segmented; (3) propodeal spines present or absent in major and minor workers; (4) eyes present or absent in major and minor workers.

##### ﻿Key to *Carebara* species of China based on major worker caste

This key is based on Xu (2003) and Zhou et al. (2006), including 34 species and subspecies; some illustrations of the key were drawn from Xu (2003) and Terayama (1996). The following species are excluded from this key because descriptions of the major workers are unavailable: *C.amia*, *C.castanea*, and *C.lignata*. Some distribution data about Chinese *Carebara* species in previous studies are ambiguous. After verification with Xu (pers. comm. July 21, 2023), some records are not accepted in this study and the following species are excluded from the Chinese fauna: *C.asina*, *C.bengalensis* (Forel, 1902), *C.bruni* (Forel, 1913), *C.cribriceps* (Wheeler, 1927), and *C.pumilia*Fischer et al., 2014.

**Table:** 

1	Antenna 11-segmented (Fig. 2A)	**2**
–	Antenna 9- or 10-segmented (Fig. 2B, C)	**17**
2	Worker caste polymorphic, with continuous series of intermediates between minor and largest major worker	**3**
–	Worker caste dimorphic	**13**
3	Propodeal spines long, > 1/2 of the distance between the base of two spines (Fig. 3A)	**4**
–	Propodeal spines short, < 1/3 of the distance between the base of 2 spines (Fig. 3B)	**9**
4	Largest major worker with mesoscutellum strongly convex in lateral view and with single ocellus on the front of head (Fig. 4A, C)	**5**
–	Largest major worker with mesoscutellum broadly convex in lateral view and with no ocelli on the front of head (Fig. 4B, D)	**7**
5	Largest major worker distinctly smaller with TL 11–12 mm	***C.diversadraco* (Santschi, 1920)**
–	Largest major worker distinctly larger with TL ~ 16 mm	**6**
6	The posterior quarter of the head with transverse and very large wrinkles; the smooth frontal space more extensive (Fig. 5A)	***C.diversalaotina* (Santschi,1920)**
–	The posterior 1/3 of head with diverged wrinkles; smooth frontal space more narrowed (Fig. 5B)	***C.diversa* (Jerdon)**
7	Propodeal spines curving forward and inclined (Fig. 6A)	***C.yanoi* (Forel)**
–	Propodeal spines directed backwards or erect (Fig. 6B)	**8**
8	Promesonotum slightly convex in lateral view; first tergite of gaster > 2× as wide as second tergite	***nanningensis* (Li & Tang)**
–	Promesonotum distinctly convex in lateral view; first gastric tergum almost as wide as second tergum	***C.affinis* (Jerdon)**
9	Head and body mostly smooth and shiny (Fig. 7A)	**10**
–	Head and body coarsely striate (Fig. 7B)	**12**
10	Propodeal spines pointing backwards in the largest major worker; petiolar node broadly rounded above in profile view; head without a coarse black line in median longitudinal groove (Fig. 8A, C)	***C.latinoda* (Zhou & Zheng)**
–	Propodeal spines curving forward in the largest major worker; petiolar node narrowed above, triangular in profile view; head with a coarse black line in median longitudinal groove (Fig. 8B, D)	**11**
11	Postpetiole approximately as long as wide; hairs sparse (Fig. 9A, C)	***C.vespillo* (Wheeler)**
–	Postpetiole distinctly broader than long; hairs abundant (Fig. 9B, D)	***C.melasolena* (Zhou & Zheng)**
12	Propodeal spines laterally compressed and curving forward; mandibles with longitudinal striations on the base in full-face view; interspaces on the head between striations punctured (Fig. 10A, C)	***C.trechideros* Zhou & Zheng**
–	Propodeal spines thick and straight, not curved; mandibles smooth in full-face view; interspaces between striations smooth (Fig. 10B, D)	***C.zengchengensis* (Zhou et al.)**
13	Mandible with 5 teeth on masticatory margin (Fig. 11A)	**14**
–	Mandible with 6 teeth on masticatory margin (Fig. 11B)	**16**
14	Posterolateral corners of head with minute tubercles; eyes present; head slightly longer than wide (Fig. 12A)	***C.altinodu* s (Xu)**
–	Posterolateral corners of head with developed horns; eyes absent; head distinctly longer than wide (Fig. 12B)	**15**
15	Head broader behind than in front with more punctures; head and thorax with dense hairs	***C.capreola* (Wheeler)**
–	The posterior portion of head almost as wide as the anterior portion; head with less punctures, smoother and more shiny; head and thorax with sparser and shorter hairs	***C.capreolalaeviceps* (Wheeler)**
16	Eyes absent; propodeal denticles downwardly inclined; first segment of gaster finely punctuate (Fig. 13A)	***C.curvispina* (Xu)**
–	Eyes present; propodeal denticles dorsoposteriorly pointed; first segment of gaster densely longitudinally striate (Fig. 13B)	***C.striata* (Xu)**
17	Mandible with 6 teeth on masticatory margin (Fig. 14C)	**18**
–	Mandible with 4 or 5 teeth on masticatory margin (Fig. 14A, B)	**19**
18	HL < 1 mm; first gastral tergum smooth and shiny	***C.oni* (Terayama)**
–	HL > 1 mm; first gastral tergum punctate and microreticulate with longitudinal striations (Fig. 15A, B)	***C.qianliyan* Terayama**
19	Posterolateral corners of head with a pair of distinct horns or small tubercles (Fig. 16A, B)	**20**
–	Head with no horns or tubercles (Fig. 16C)	**29**
20	Propodeum with a pair of protruding denticles (Fig. 17A)	**21**
–	Propodeum without a pair of protruding denticles; posterodorsal corner of propodeum rounded or forms an obtuse or right angle (Fig. 17B, C)	**23**
21	Posterior area of head without transverse striations; metanotal groove impressed shallowly; body smaller with TL 1.4 mm (Fig. 18C)	***C.acutispina* (Xu)**
–	Posterior area of head with transverse striations; metanotal groove deeply impressed; body larger with TL 2.1–2.6 mm (Fig. 18A, B)	**22**
22	Mandible with 4 teeth on masticatory margin; clypeus with the anterior margin of median portion concave indistinctly	***wheeleri* (Ettershank)**
–	Mandible with 5 teeth on masticatory margin; clypeus with the anterior margin of median portion concave distinctly	***C.obtusidenta* (Xu)**
23	Horns connected by a developed transverse ridge (Fig. 19A)	**24**
–	Horns not connected by a developed transverse ridge (Fig. 19B)	**25**
24	Head capsule thin with straight anterior margin in lateral view (Fig. 20A)	***C.bihornata* (Xu)**
–	Head capsule thick with convex anterior margin in lateral view (Fig. 20B)	***C.sakamotoi* Terayama et al.**
25	Body larger with TL 3.0–3.5 mm	***C.polyphemus* (Wheeler)**
–	Body smaller with TL 1.5–2.3 mm	**26**
26	Mandible with 4 teeth on masticatory margin	***C.taiponica* (Wheeler)**
–	Mandible with 5 teeth on masticatory margin	**27**
27	Head coarsely microreticulate; frons and vertex with many striae (Fig. 21A)	***C.yamatonis* (Terayama)**
–	Head largely smooth and shiny (Fig. 21B)	**28**
28	Anterodorsal corner of propodeum prominent, forming an acute tooth behind metanotal groove (Fig. 22A)	***C.sauteri* (Forel)**
–	Anterodorsal corner of propodeum not forming an acute tooth (Fig. 22B)	***C.rectidorsa* (Xu)**
29	Head nearly square, ~ as long as broad; eyes with 16 facets; head with 3 ocelli; dorsum of mesosoma straight (Fig. 23A, B)	***C.hunanensis* (Xu)**
–	Head rectangular, longer than broad; eyes with < 10 facets; head without ocelli; dorsum of mesosoma not straight	**30**
30	Propodeum with a pair of acute teeth; head with fine reticulations	***C.reticapita* (Xu)**
–	Propodeum forms an obtuse angle; head smooth, at most sparsely punctured	**31**
31	Posterodorsal corner of propodeum forming a right angle of ~ 90° (Fig. 24A)	***C.pseudolusciosa* (Wu & Wang)**
–	Posterodorsal corner of propodeum forming an obtuse angle of more than 90° (Fig. 24B)	**32**
32	Vertex with transverse striations	***C.jiangxiensis* (Wu & Wang)**
–	Vertex smooth and with no striations	**33**
33	Antenna 10-segmented; katepisternum rugose-reticulate; body distinctly larger with TL > 2.6 mm (Fig. 25A)	***C.laeviceps* sp. nov.**
–	Antenna 9-segmented; katepisternum smooth and shiny; body smaller with TL ~ 2 mm (Fig. 25B)	***C.lusciosa* (Wheeler)**

**Figure 2.**
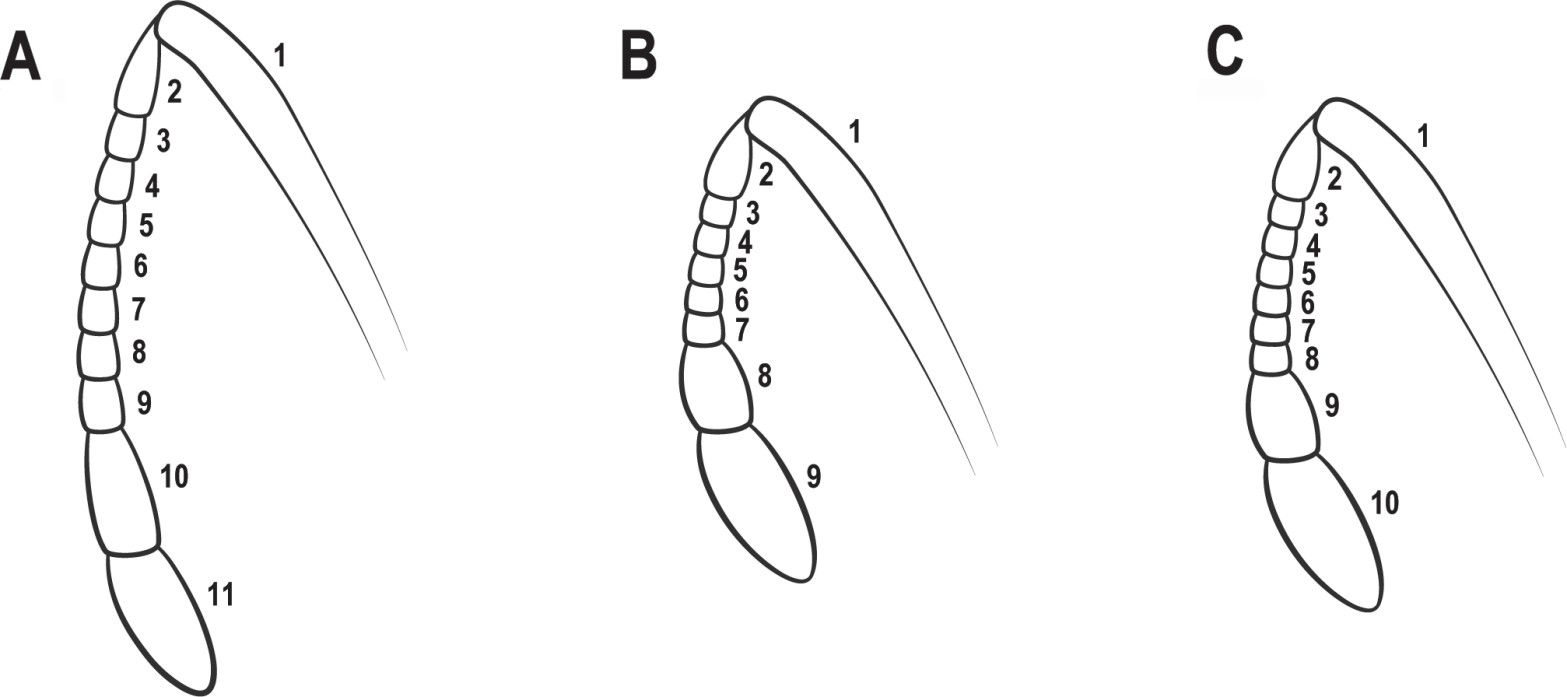
Antenna **A** 11-segmented **B** 9-segmented **C** 10-segmented.

**Figure 3.**
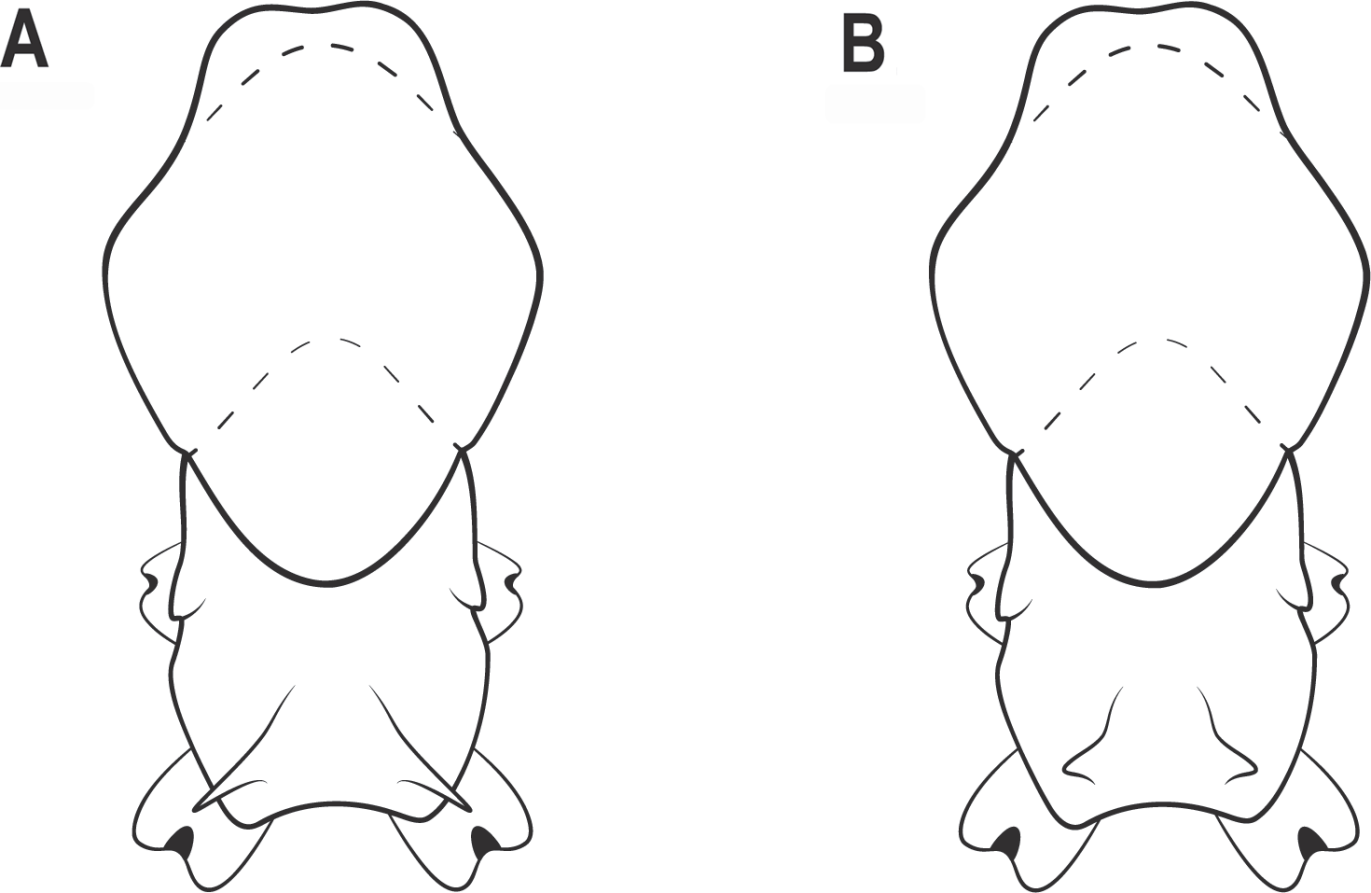
Mesosoma **A** longer spine **B** shorter spine.

**Figure 4.**
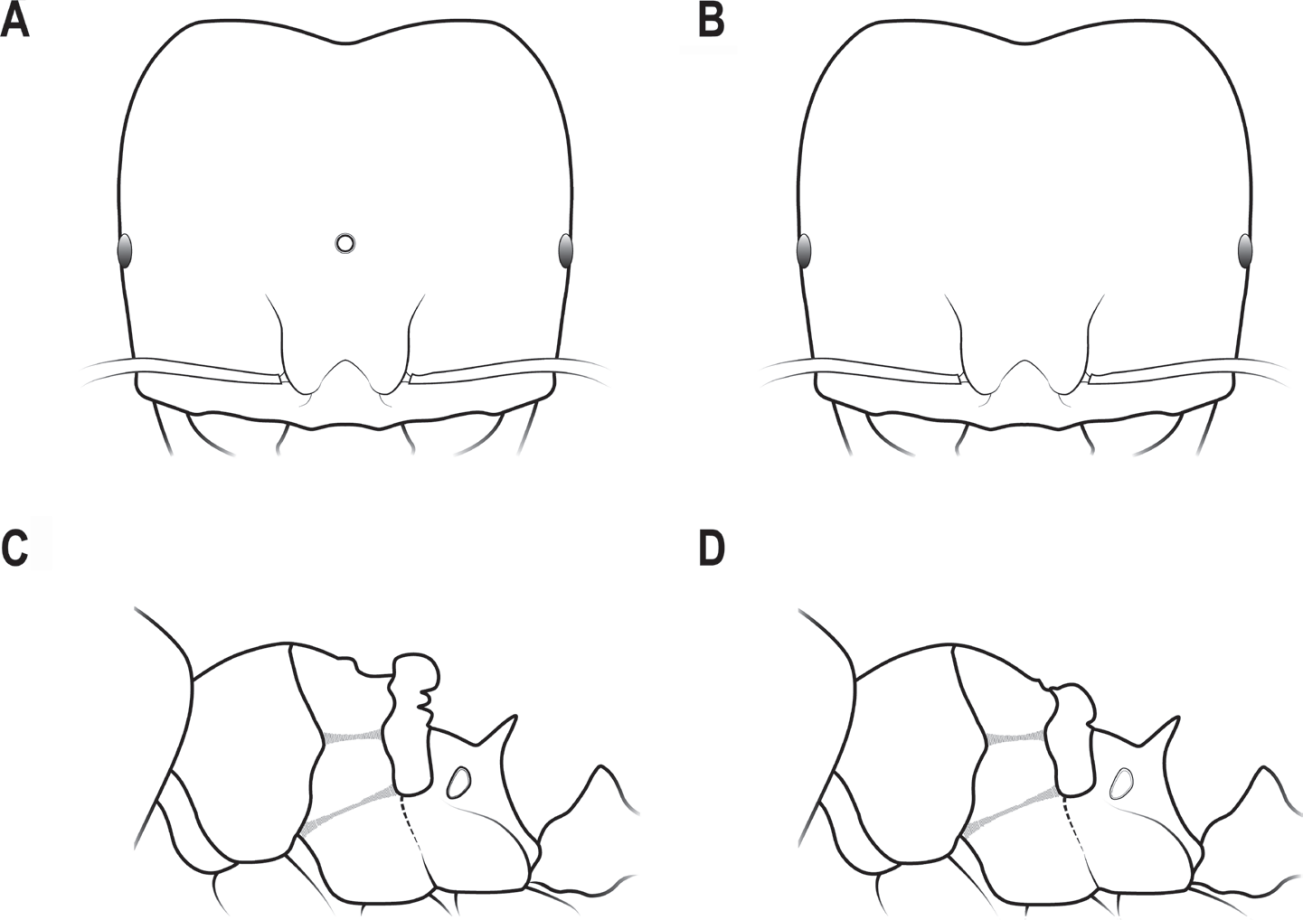
Head in full-face view and mesosoma in lateral view (the largest major worker) **A** head with an ocellus **B** head with no ocelli **C** mesoscutellum strongly convex **D** mesoscutellum broadly convex.

**Figure 5.**
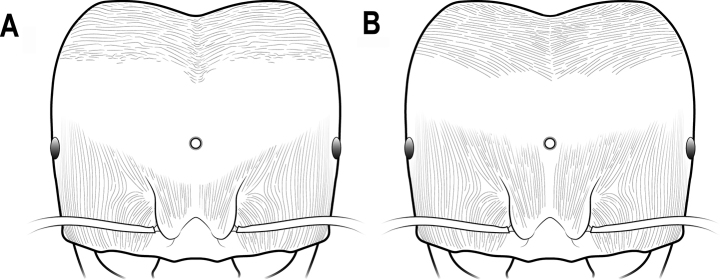
Heads of *C.diversa* and *C.diversalaotina* in full-face view (the largest major worker) **A***C.diversalaotina*, head with broader smooth space, wrinkles mostly transverse **B***C.diversa*, head with more narrowed smooth space, wrinkles fine and diverged.

**Figure 6.**
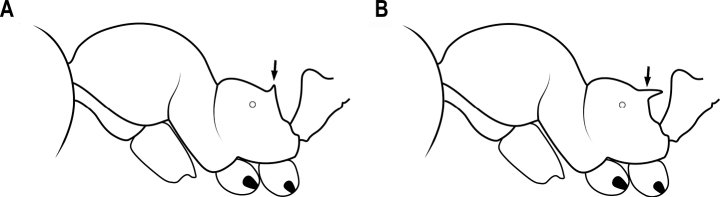
Pronotal spines **A** curving forward **B** backwards.

**Figure 7.**
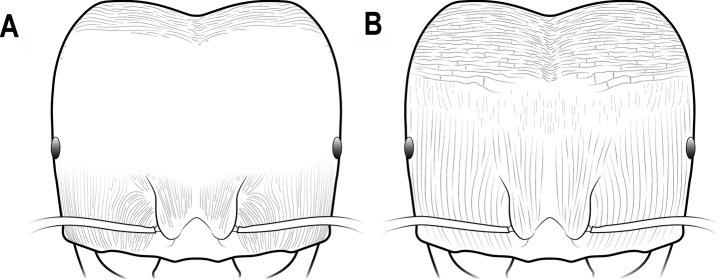
Head in full-face view **A** head mostly smooth and shiny **B** head mostly striate.

**Figure 8.**
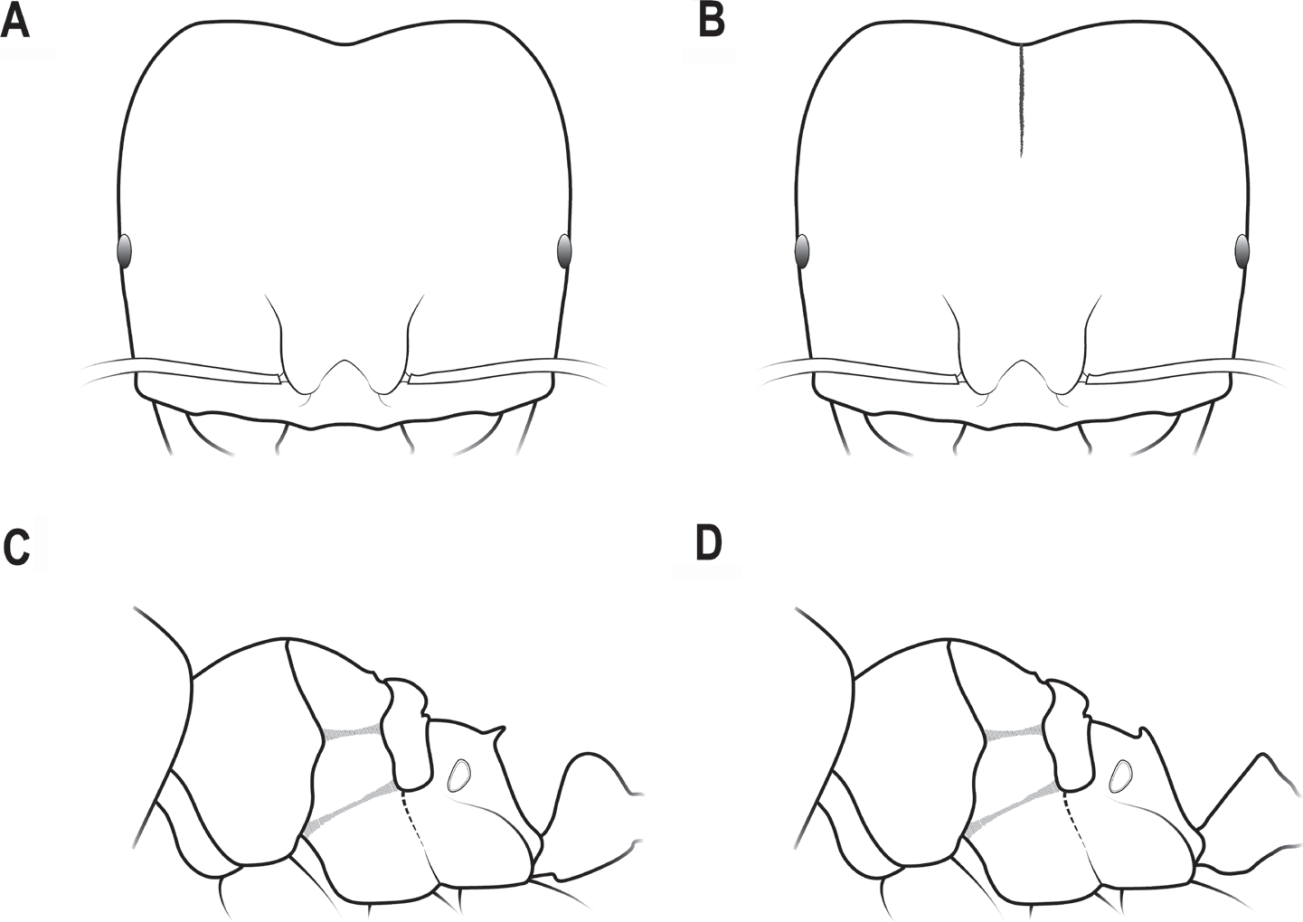
Head in full-face view, mesosoma and petiole in lateral view **A** head without a coarse black line **B** head with a coarse black line in median longitudinal groove **C** propodeal spines pointing backward, petiolar node round above **D** propodeal spines curving forward, petiolar node narrowed above.

**Figure 9.**
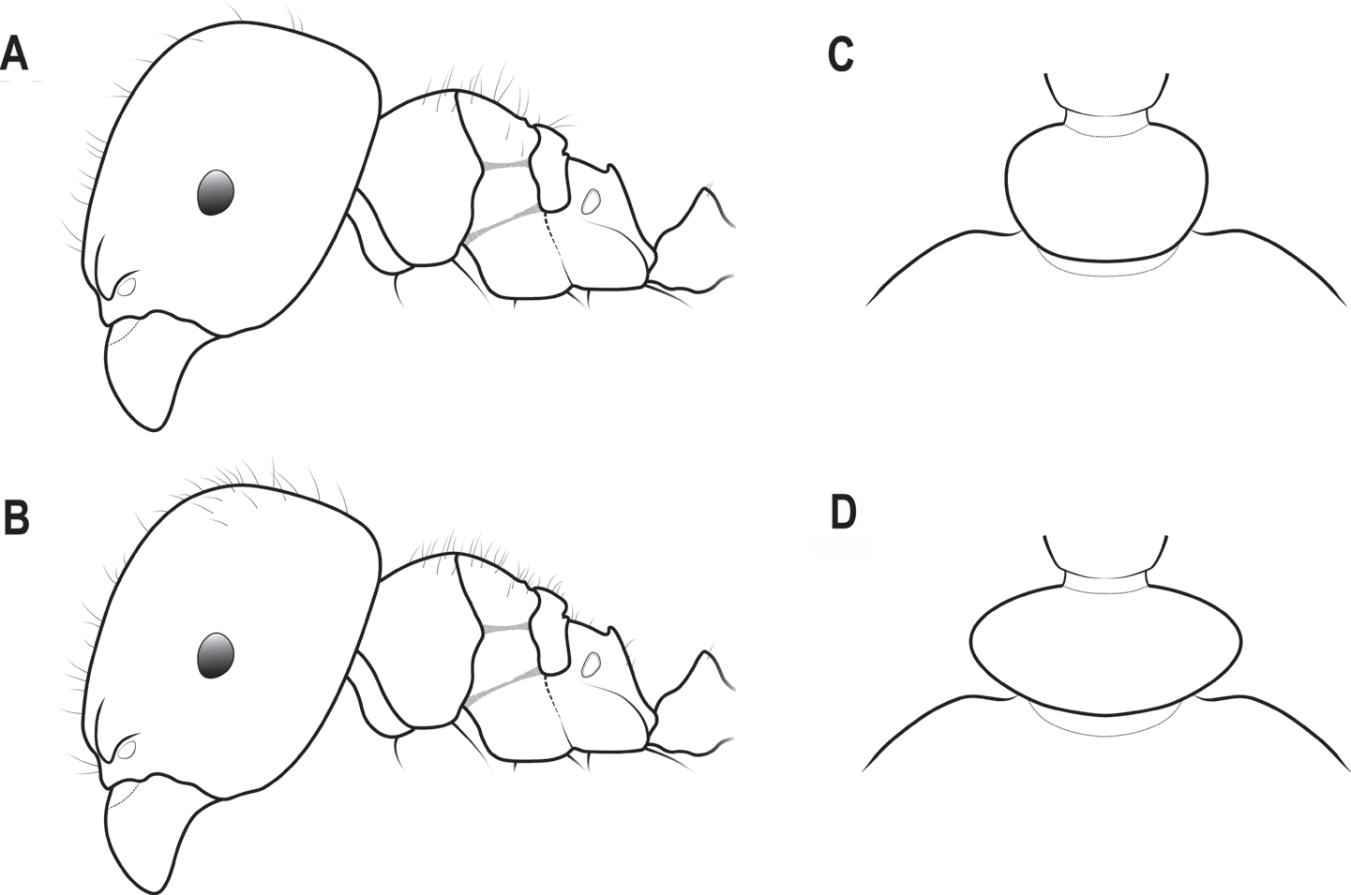
Head and mesosoma in lateral view, postpetiolar node in dorsal view **A***C.vespillo*, hairs sparse **B***C.melasolena*, hairs abundant **C***C.vespillo*, postpetiolar node nearly as long as wide **D***C.melasolena*, postpetiolar node distinctly wider than long.

**Figure 10.**
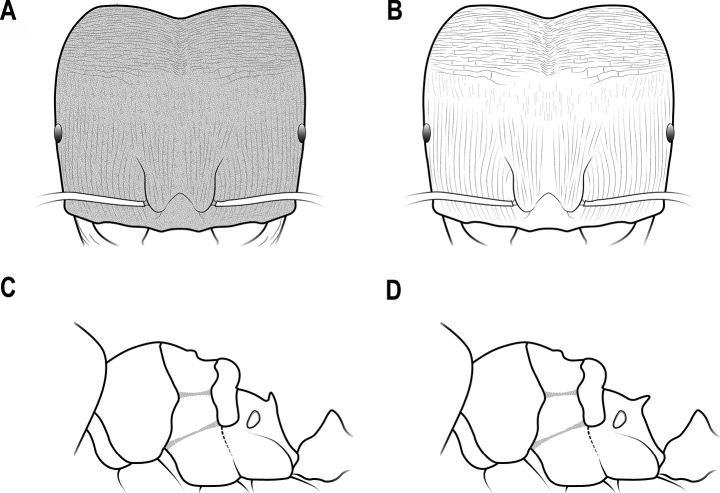
Head in full-face view and mesosoma in lateral view (the largest major worker) **A** head of *C.trechideros*, mandibles with longitudinal striations, interspaces between striations punctured **B** head of *C.zengchengensis*, mandibles without striations, interspaces between striations smooth **C** propodeal spines curving forward **D** propodeal spines straight and not curved.

**Figure 11.**
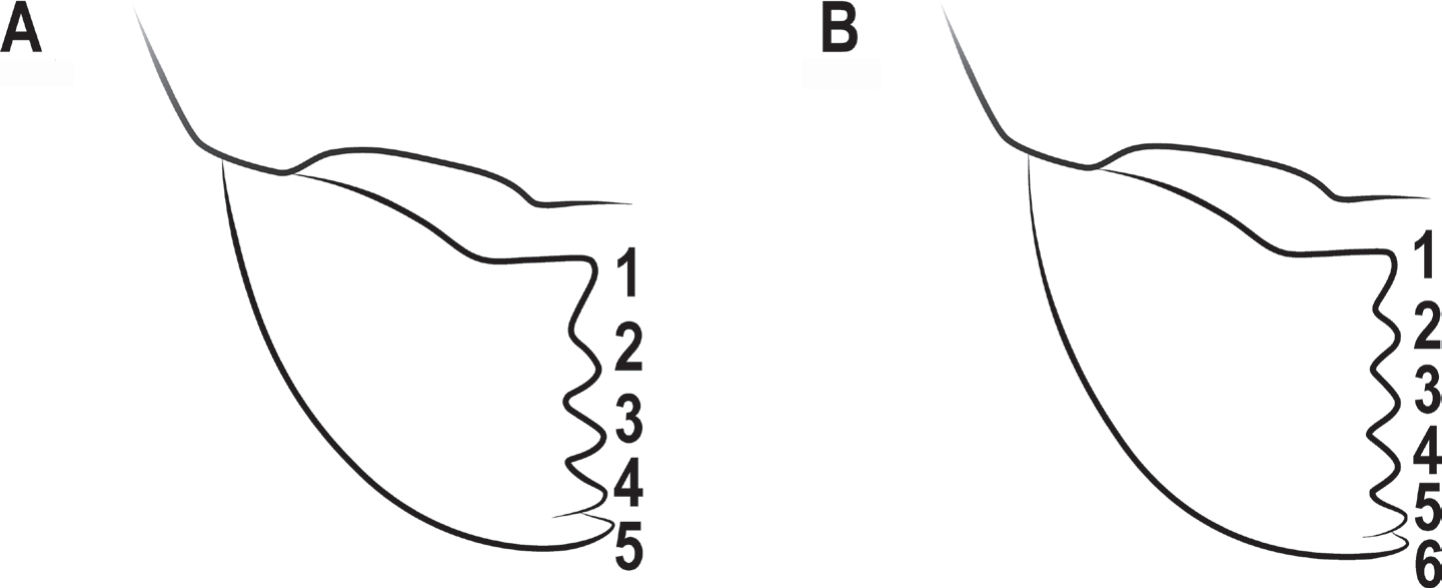
Mandibles **A** mandible with 5 teeth **B** mandible with 6 teeth.

**Figure 12.**
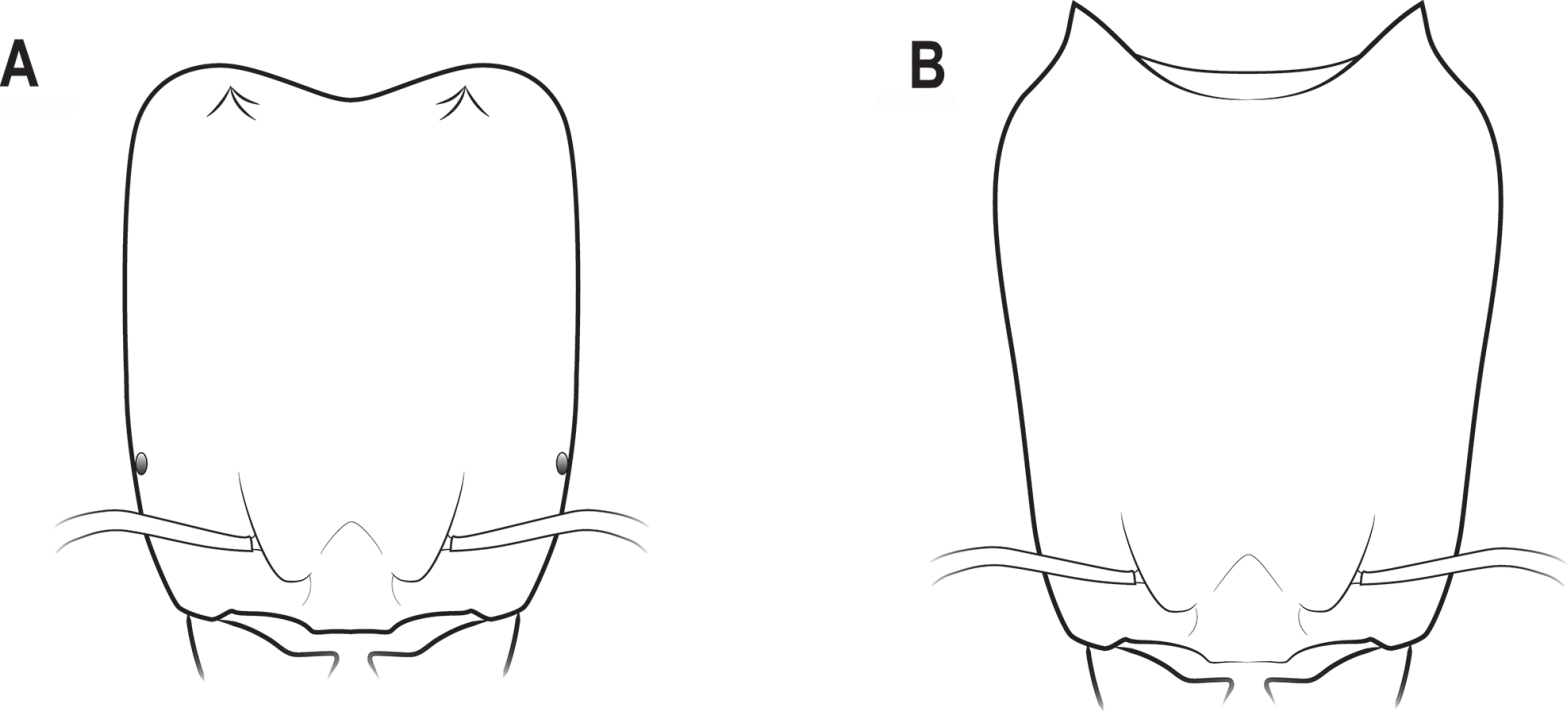
Horns **A** posterolateral corners of head with minute tubercles **B** posterolateral corners of head with developed horns.

**Figure 13.**
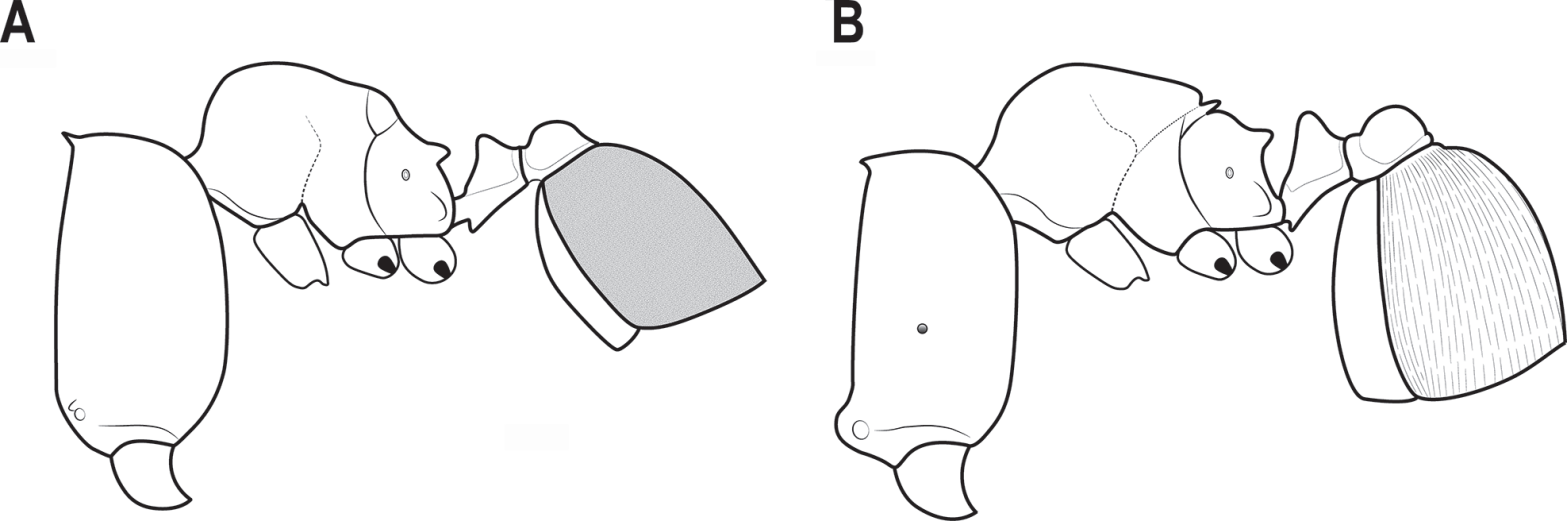
Two *Carebara* species in lateral view **A***C.curvispina*, propodeal denticles down-inclined, first segment of gaster finely punctuate, eyes absent **B***C.striata*, propodeal denticles dorsoposteriorly pointed, first segment of gaster densely longitudinally striate, eyes present.

**Figure 14.**
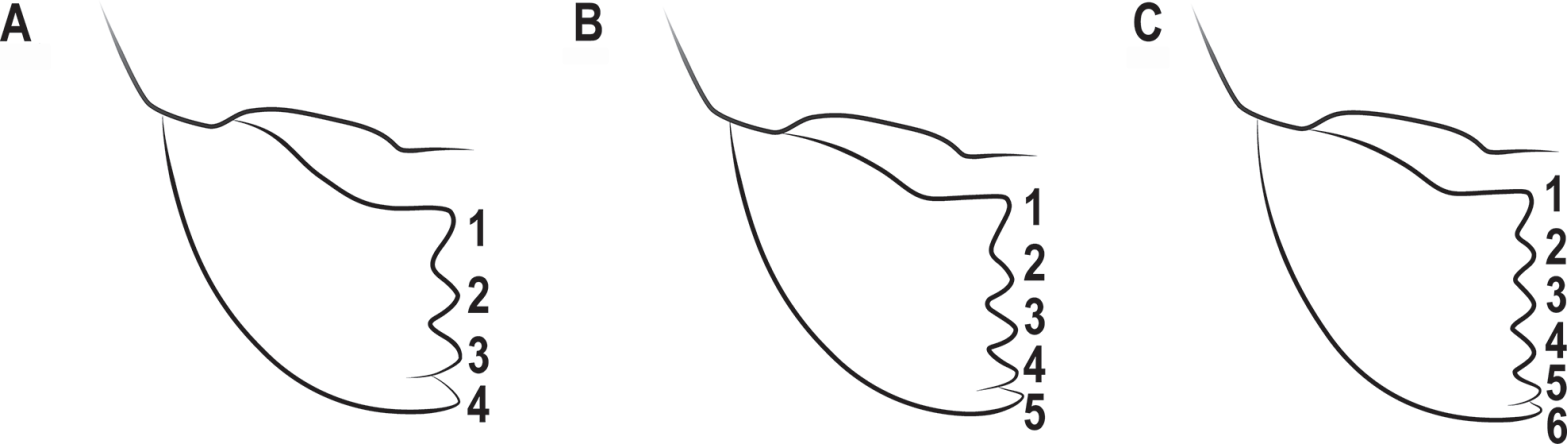
The number of teeth **A** 4-teethed **B** 5-teethed **C** 6-teethed.

**Figure 15.**
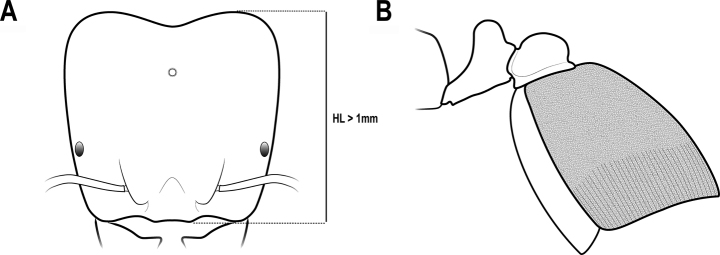
Head in full-face view and tergum in lateral view (*C.qianliyan*) **A**HL > 1 mm **B** first gastral tergum punctate with longitudinal striations.

**Figure 16.**
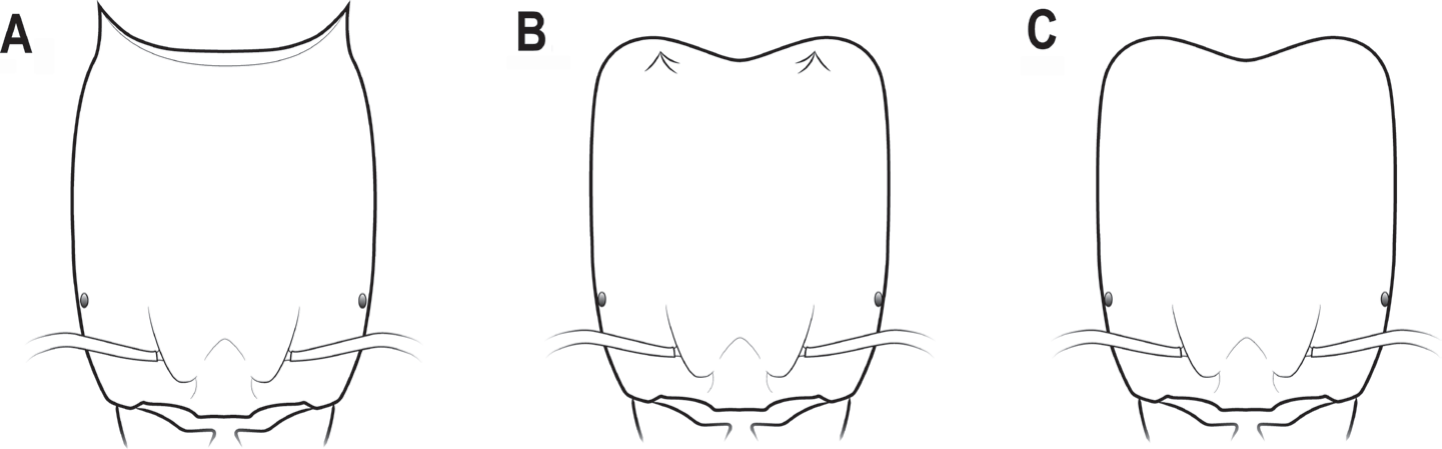
Horns or tubercles on the posterior corner of head **A** distinct horns **B** small tubercles **C** no horns or tubercles.

**Figure 17.**
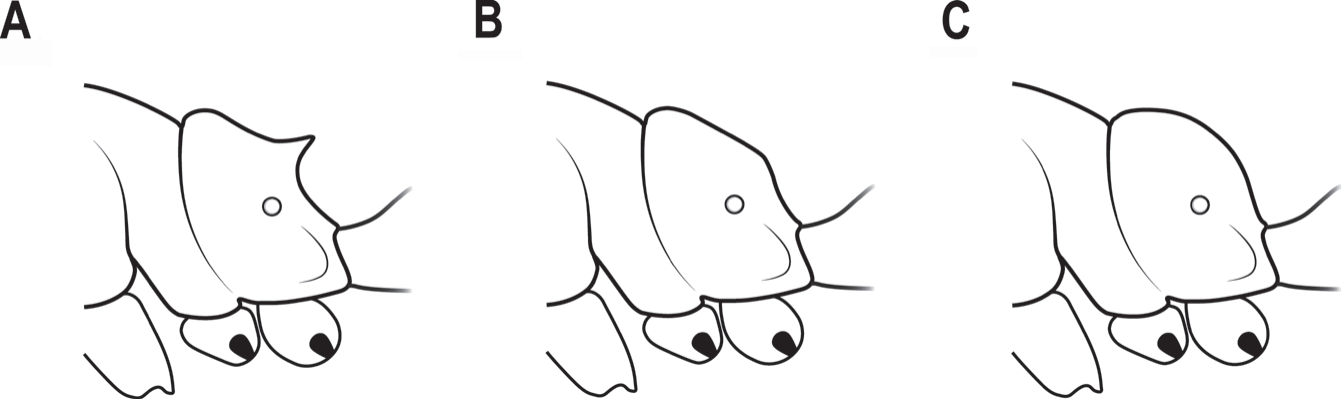
Propodeum in lateral view **A** propodeum with protruding denticles **B** propodeum forming an obtuse angle **C** propodeum rounded.

**Figure 18.**
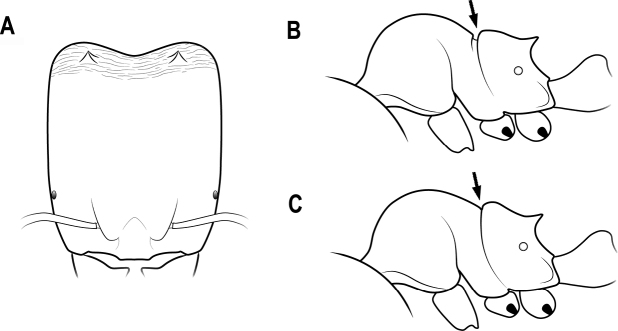
Striations and metanotal groove **A** head with transverse striations **B** metanotal groove impressed deeply **C** metanotal groove impressed shallowly.

**Figure 19.**
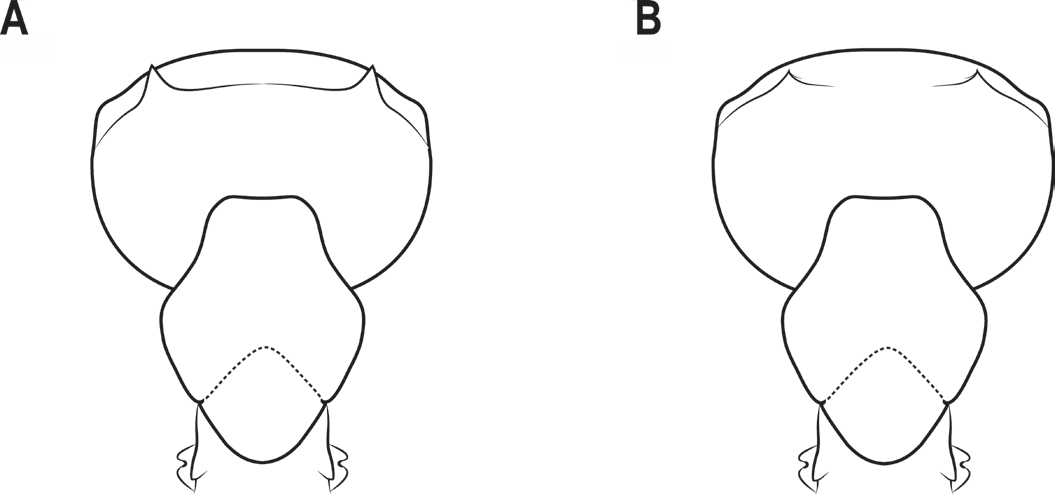
Horns in dorsal view **A** horns connected by a transverse ridge **B** horns not connected by a transverse ridge.

**Figure 20.**
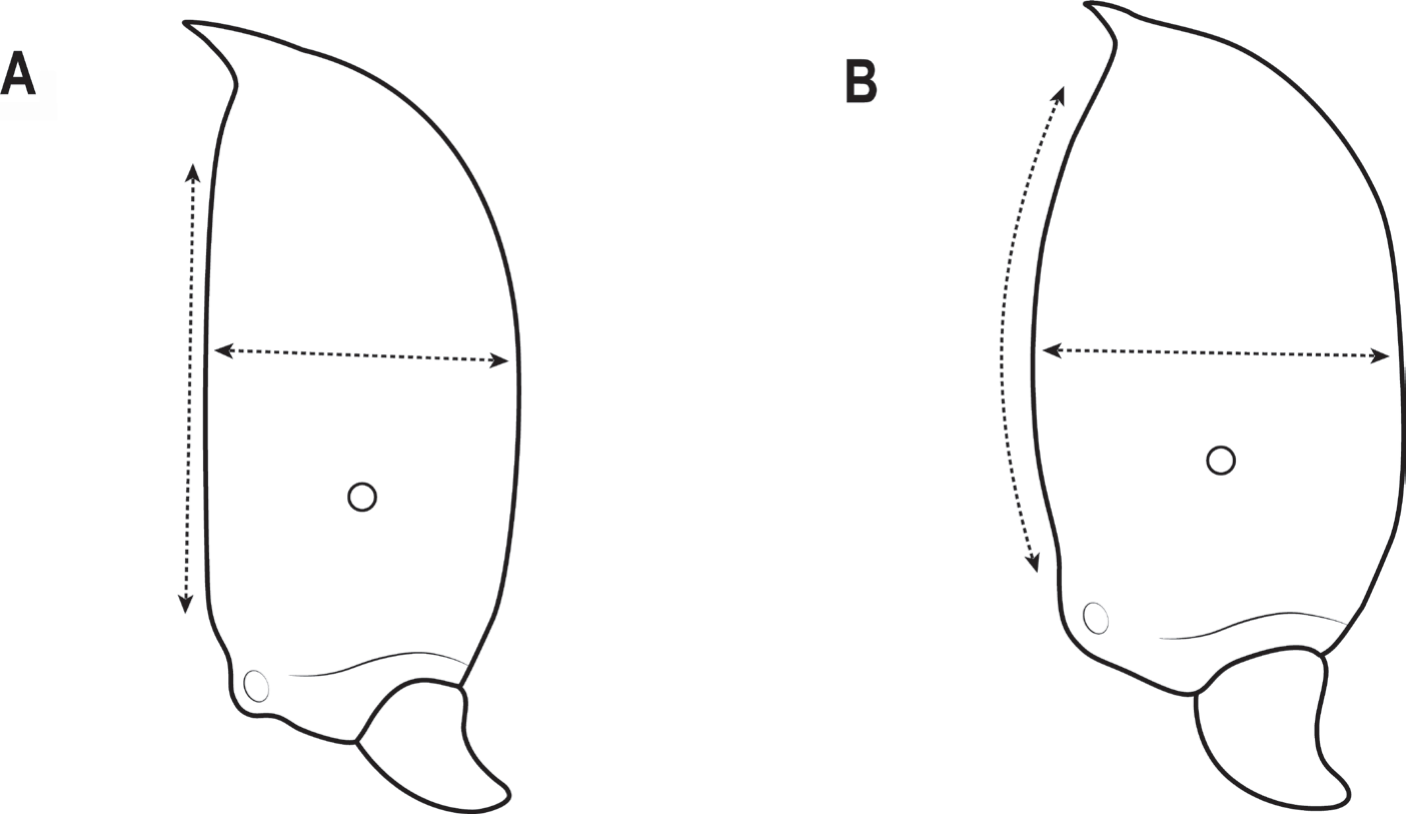
Head capsule in lateral view **A** head capsule thin with anterior margin straight **B** head capsule thick with anterior margin convex.

**Figure 21.**
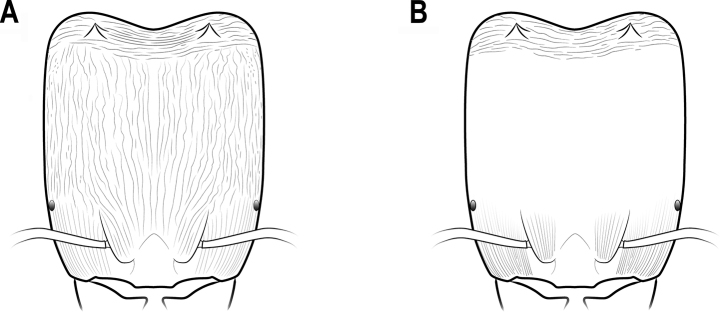
Head in full-face view **A** head with dense microreticulation **B** head largely smooth and shiny.

**Figure 22.**
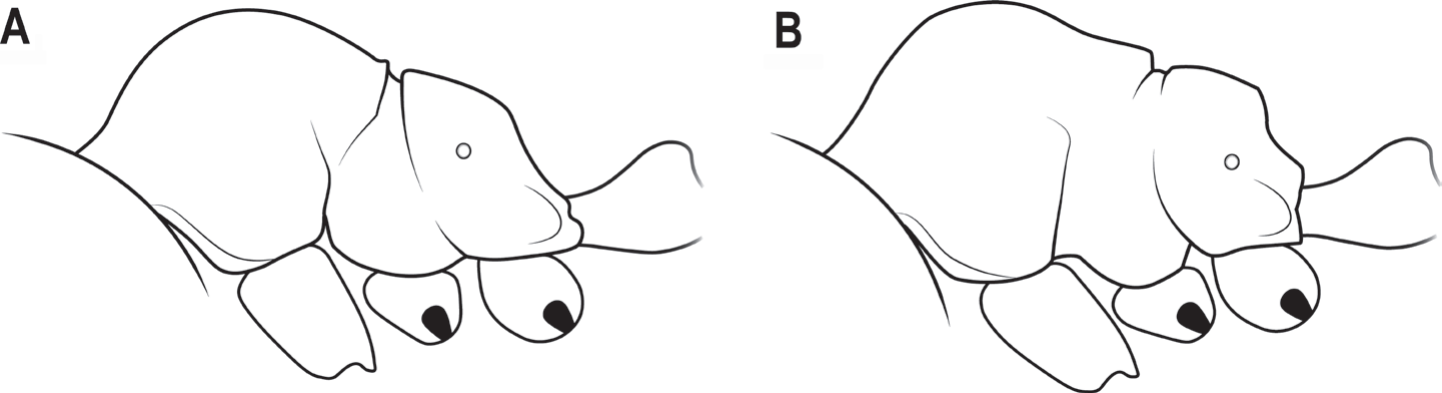
Mesosoma in lateral view **A** anterodorsal corner of propodeum forming an acute tooth **B** anterodorsal corner of propodeum not forming an acute tooth.

**Figure 23.**
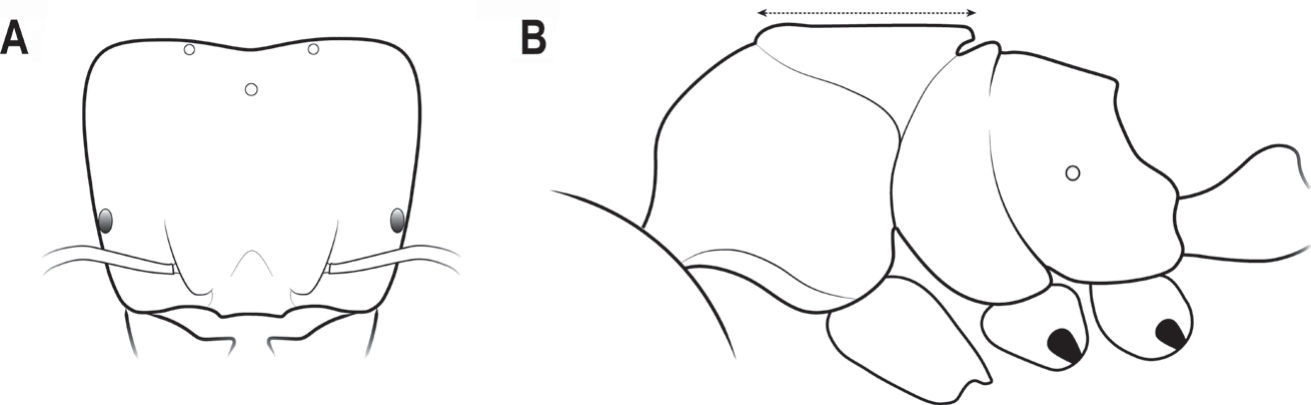
*C.hunanensis***A** head with 3 ocelli in full-face view **B** dorsum of mesosoma straight in lateral view.

**Figure 24.**
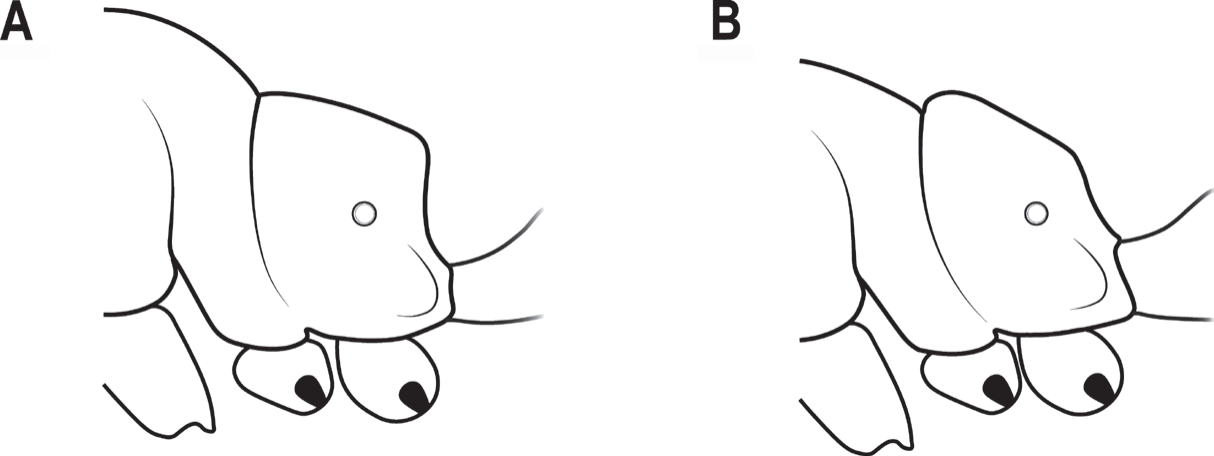
Propodeum in lateral view **A** posterodorsal corner forming an right angle **B** posterodorsal corner forming an obtuse angle.

**Figure 25.**
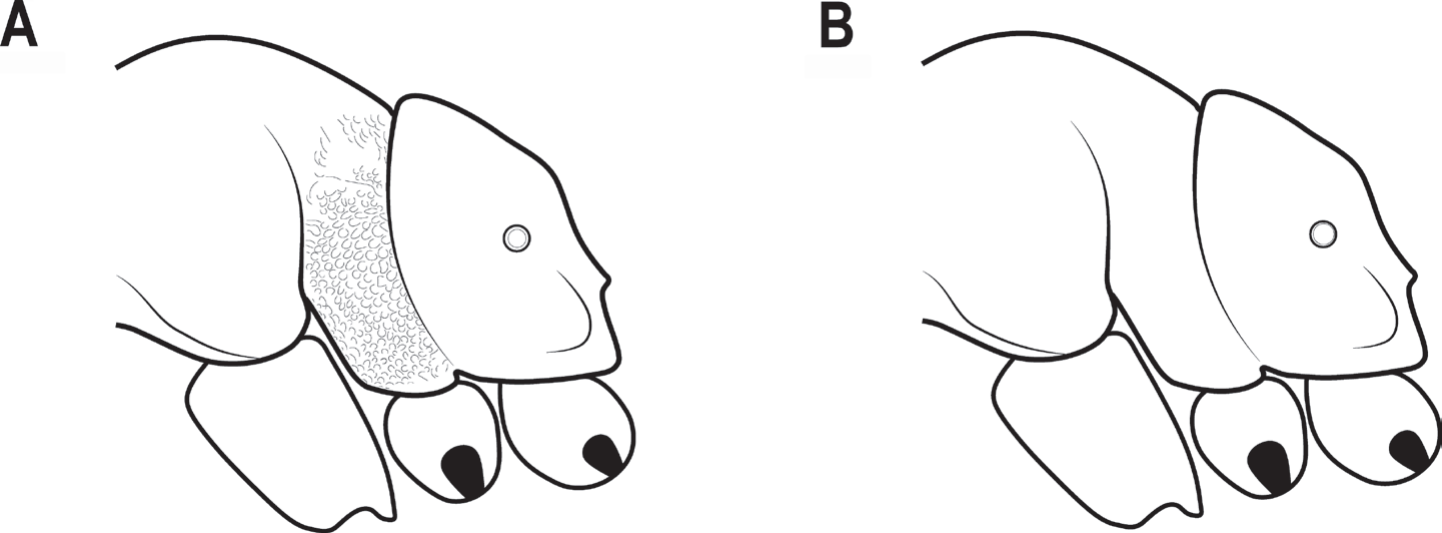
Katepisternum in lateral view **A** katepisternum rugose-reticulate **B** katepisternum smooth.

### ﻿ Carebara laeviceps

Taxon classificationAnimaliaHymenopteraFormicidae

Liu & Zhong sp. nov.

F79A4277-8CC6-506B-89A7-93537222F769

https://zoobank.org/A584356D-EADC-4344-9142-4F34D030F4DE

**Figure 26.**
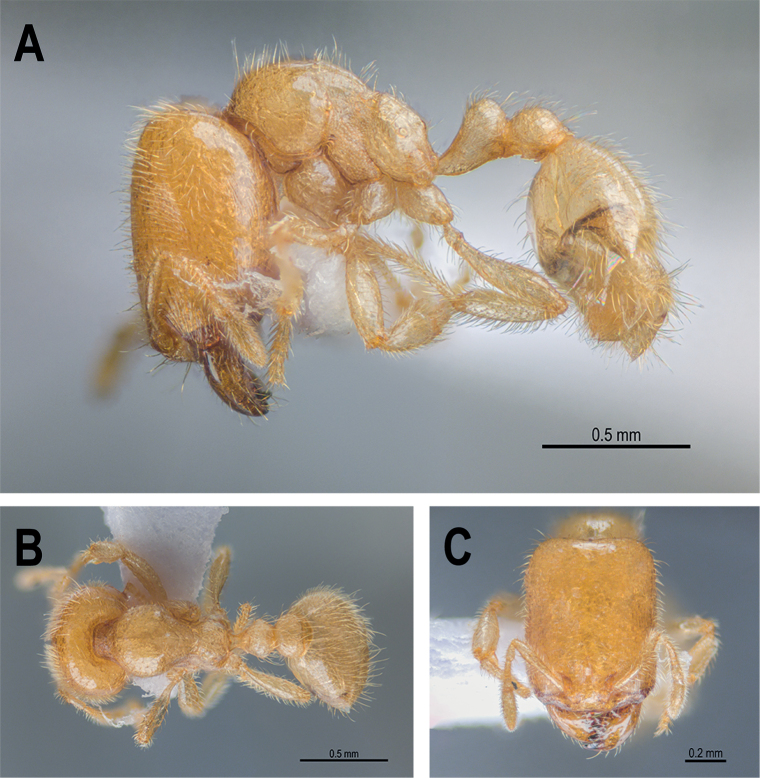
*Carebaralaeviceps* Liu & Zhong, sp. nov. Holotype (major worker) **A** body in lateral view **B** body in dorsal view **C** head in full-face view.

**Figure 27.**
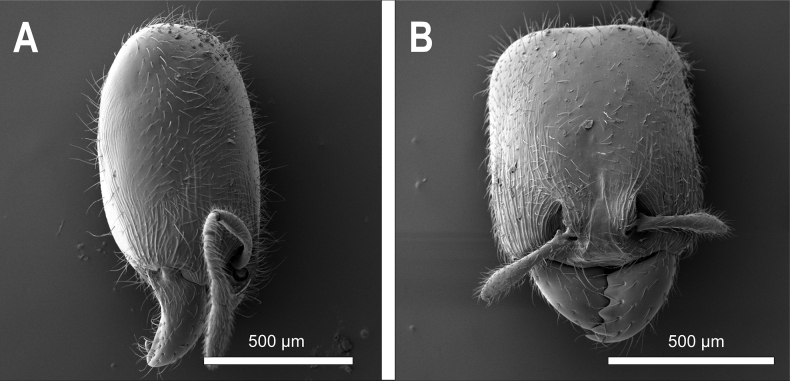
*Carebaralaeviceps* Liu & Zhong, sp. nov. Major worker under SEM (not holotype) **A** head in lateral view **B** head in full-face view.

**Figure 28.**
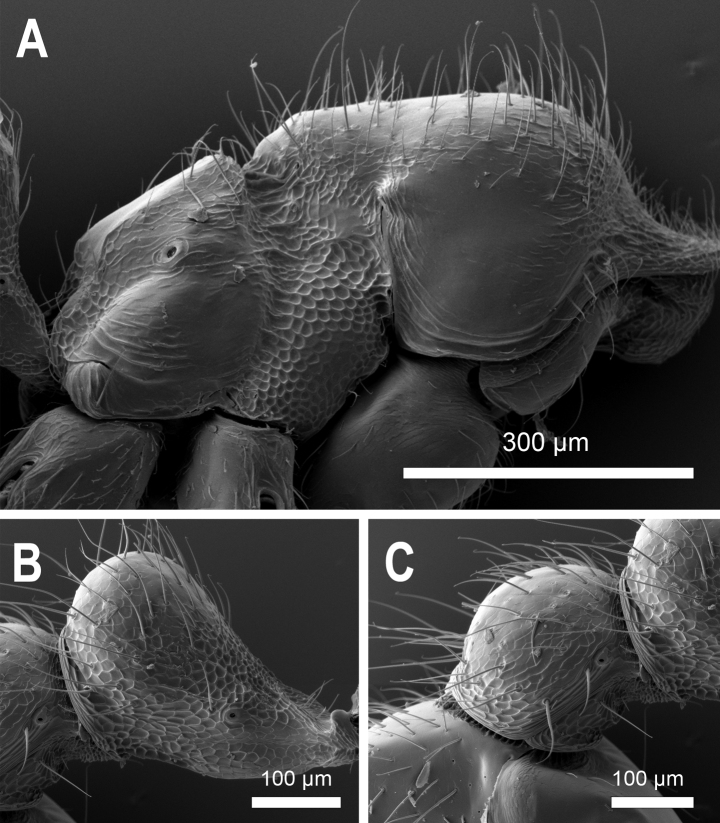
*Carebaralaeviceps* Liu & Zhong, sp. nov. Major worker under SEM (not holotype) **A** mesosoma in lateral view **B** petiole in lateral view **C** postpetiole in lateral view.

**Figure 29.**
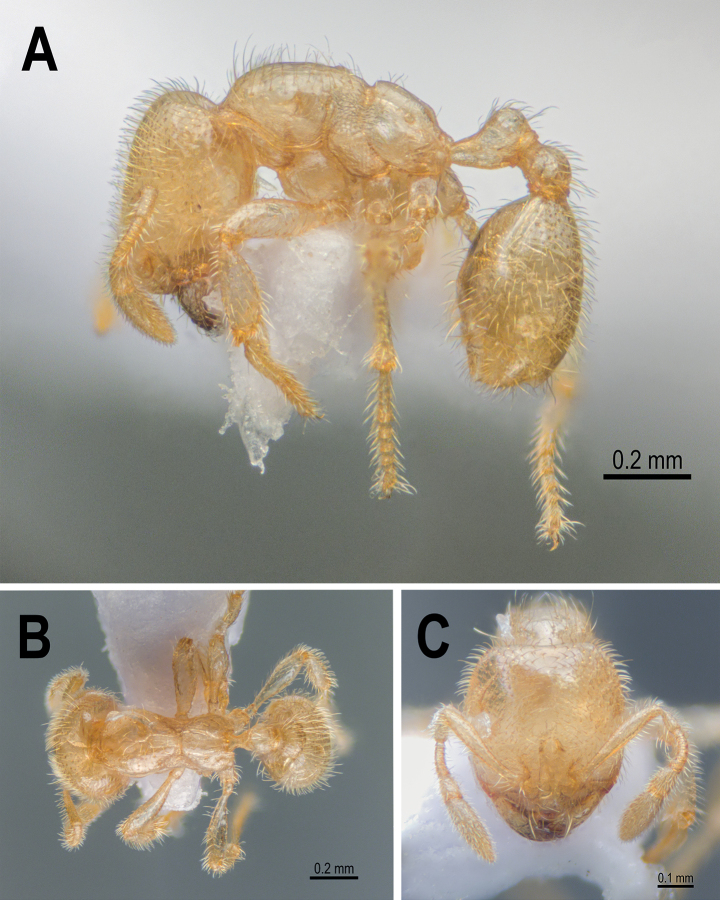
*Carebaralaeviceps* Liu & Zhong, sp. nov. Paratype minor worker **A** body in lateral view **B** body in dorsal view **C** head in full-face view.

**Figure 30.**
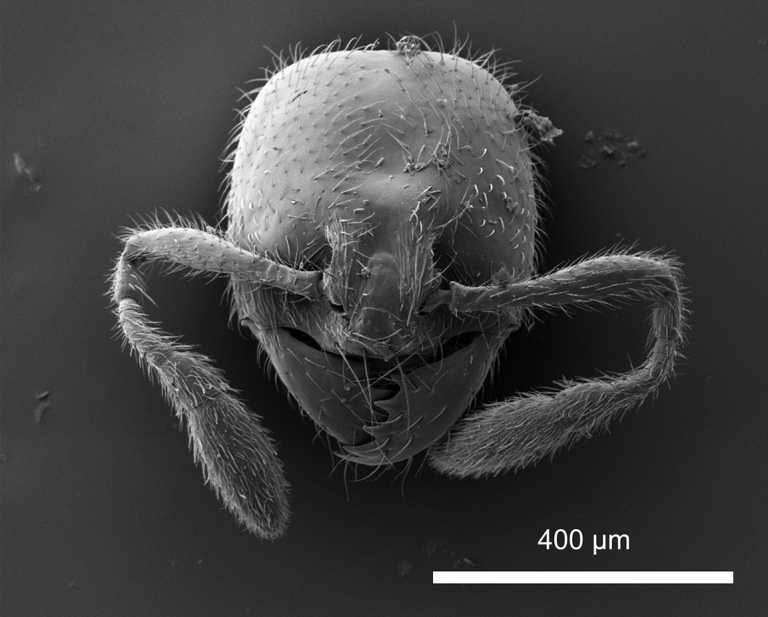
*Carebaralaeviceps* Liu & Zhong, sp. nov. Minor worker under SEM. head in full-face view.

**Figure 31.**
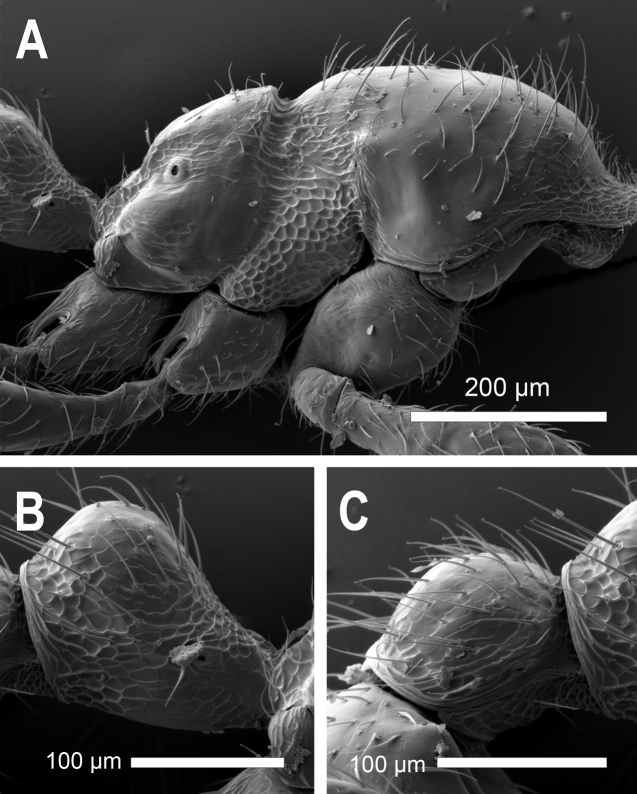
*Carebaralaeviceps* Liu & Zhong, sp. nov. Minor worker under SEM **A** body in lateral view **B** petiole in lateral view **C** postpetiole in lateral view.

Figs 26 , 27 , 28 , 29 , 30 , 31

#### Type material.

SWFU; ***Holotype*.** China: 1 major worker, Sichuan Province, Dazhou City, Kaijiang County, 31°12'24"N, 107°55'43"E, alt. 1100 m, 27.VI.2022, Gui-chuan Nie, SWFU A22-955. ***Paratypes*.** China: 3 major workers and 4 minor workers, same data as holotype, SWFU A22-955.

#### Description of major worker.

***Measurements*.** Holotype major worker: HL 0.84, HW 0.63, EL 0.02, SL 0.38, WL 0.73, PNW 0.42, PNH 0.29, MNH 0.48, PDH 0.32, PTL 0.30, PTH 0.23, PTW 0.22, PPL 0.21, PPH 0.18, PPW 0.25, CI 75, SI 60, EI 3, LPpI 117, DPpI 119, PpWI 114, PpLI 70, PpHI 78, PPI 60. Paratype major workers (*n* = 3): HL 0.88 (0.85–0.90), HW 0.68 (0.66–0.70), EL 0.03 (0.02–0.05), SL 0.36 (0.35–0.36), WL 0.76 (0.75–0.78), PNW 0.43 (0.42–0.44), PNH 0.33 (0.31–0.35), MNH 0.44 (0.43–0.44), PDH 0.33 (0.30–0.35), PTL 0.30 (0.26–0.33), PTH 0.23 (0.22–0.23), PTW 0.20 (0.19–0.20), PPL 0.17 (0.14–0.19), PPH 0.18 (0.15–0.20), PPW 0.26 (0.25–0.27), CI 78 (77–78), SI 52 (51–55), EI 4.38 (3–7.14), LPpI 107 (94–127), DPpI 144 (131–153), PpWI 132 (130–135), PpLI 59 (57–61), PpHI 75 (65–83), PPI 60 (58–61). ***Head*.** Large, subrectangular with lateral margins straight and parallel in full-face view, distinctly longer than broad, ~ 1.3× as long as wide; posterior margin slightly concave medially; posterolateral corner rounded and without horns in lateral view. Mandible triangular with five teeth on the masticatory margin. Clypeus steep and flat in lateral view; anterior margin of clypeus nearly straight with median portion indistinctly concave. Frontal lobes concealing condylar bulb. Ocelli absent. Eyes minute, located a little behind the anterior 1/3 length of head, ~ 0.3 mm from mandibular insertions to eyes. Antenna 10-segmented with a 2-segmented club; scape short, ~ 0.4× as long as HL; apex of scape below mid-length of distance from antennal insertion to vertexal corner when scape is laid back. Dorsum of head flat in lateral view. ***Mesosoma*.** In lateral view, promesonotum slightly convex with moderately rounded dorsum; the sides of pronotum strongly convex and rounded in dorsal view; promesonotal suture indistinct. Metanotal groove deeply impressed. Anterodorsal corner of propodeum forms an acute tooth behind the metanotal groove in lateral view; propodeum lower than promesonotum with flat dorsum; the declivity and dorsum of propodeum forming an obtuse angle in lateral view; declivitous edge of propodeum with a pair of indistinct carinae; lateral margins of propodeum strongly convex in dorsal view. ***Waist*.** Petiole ~ 0.8× as high as long with a long peduncle; petiolar node wider than long in dorsal view. In lateral view, the peduncle without angled tooth in anteroventral corner and the ventral margin of peduncle slightly convex; dorsum of petiole rounded in lateral view; anterior and posterior surfaces of petiolar node moderately convex. Postpetiolar node slightly lower than petiolar node, roundly convex. In dorsal view, postpetiole wider than petiole (PPW 0.25, PTW 0.22), both petiolar and postpetiolar nodes with convex lateral margins. ***Gaster*.** Long and oval. ***Sculpture and hairs*.** Mandibles, Median portion of clypeus and area from frons to posterior margin of head smooth and shiny, except genae and frontal lobes longitudinally striate. Posterior area of head without striations or carinae. Dorsum and lateral face of pronotum mostly smooth and shiny; anterior face of pronotal disc with fine reticular rugae. Mesonotum smooth; anepisternum and katepisternum strongly rugose-reticulate. In dorsal view, metanotal groove with several longitudinally parallel rugulae; propodeum mostly smooth in dorsal view; lateral face and declivity of propodeum weakly rugose-reticulate and with indistinct transverse rugulae in lateral view. Dorsum of petiolar node smooth; the lateral faces of node and peduncle rugose-reticulate; postpetiole weakly reticulate in dorsal view; ventral area of petiole and postpetiole strongly reticulate in lateral view. Gaster smooth and shiny. Head capsule covered with erect to subdecumbent hairs; while hairs on scapes and mandibles mostly decumbent. Dorsum of pronotum and mesonotum with abundant long erect hairs in lateral view; hairs on lateral face of mesosoma and dorsum of propodeum much sparser. Dorsum of petiole and postpetiole, and gaster with long erect to decumbent hairs; the ventral margin of petiole and postpetiole with no hairs in lateral view. ***Color.*** Head yellowish brown with clypeus and genae slightly darker; masticatory margin of mandible black. Mesosoma and petiole yellowish brown. Color of appendages and gaster paler.

#### Description of minor worker.

***Measurements*.** Paratype minor workers (*n* = 4): HL 0.46 (0.44–0.48), HW 0.44 (0.42–0.46), EL 0.01, SL 0.31 (0.30–0.32), WL 0.52 (0.51–0.52), PNW 0.28 (0.27–0.29), PNH 0.20 (0.20–0.21), MNH 0.29 (0.27–0.31), PDH 0.21 (0.19–0.23), PTL 0.18 (0.17–0.19), PTH 0.15 (0.14–0.15), PTW 0.13 (0.12–0.13), PPL 0.12 (0.11–0.12), PPH 0.11 (0.10–0.11), PPW 0.16, CI 94 (88–98), SI 70 (65–74), EI 2, LPpI 110 (100–120), DPpI 139 (133–145), PpWI 128 (123–133), PpLI 64 (61–71), PpHI 73 (67–79), PPI 57 (55–59). ***Head*.** Much smaller (HL 0.44–0.48, HW 0.42–0.46) than the head of major worker. In full-face view head subquadrate with lateral margins convex, slightly longer than broad and narrowed both anteriorly and posteriorly, ~ 1.1× as long as wide. Posterior margin of head slightly concave medially, posterolateral corners rounded in full-face view. Dorsum of head broadly convex in lateral view. Anterior margin of clypeus almost straight. Mandible triangular with five teeth on masticatory margin. Eyes minute, situated at the anterior 1/2 length of head, ~ 0.2 mm from mandibular insertions to eyes. Antenna 10-segmented with a 2-segmented club; scape 0.70× as long as HW; apex of scape reaching 3/5 of the distance from antennal insertion to vertexal corner when scape is laid back. Dorsum of head broadly convex in lateral view. ***Mesosoma*.** Promesonotum with dorsal profile slightly arched in lateral view, nearly flat; suture indistinct. Metanotum absent; metanotal groove distinct and strongly impressed; In lateral view, propodeum spineless; the dorsal face of propodeum straight, forming an obtuse angle with the declivity of propodeum; declivity nearly straight, with median portion slightly concave; anterodorsal corner forming an acute tooth behind metanotal groove in lateral view. ***Waist*.** Petiole longer than high with long peduncle (PTL 0.18, PTH 0.15) in lateral view; ventral margin of petiole slightly convex; petiolar node broader than long with anterodorsal and posterodorsal faces convex in dorsal view. In lateral view, combined profile of anterior face of node and peduncle convex distinctly. Declivity of the posterior face of petiole slightly steeper than anterior face. Postpetiole with lower node than petiole, both dorsa of petiolar and postpetiolar nodes roundly convex. ***Gaster*.** Oval, relatively short. ***Sculpture and hairs*.** In full-face view, head capsule, clypeus, and mandibles mostly smooth; only gena and frontal lobe with indistinct longitudinal rugulae; sculpture on mesosoma same as major workers. Gaster smooth and shiny. Whole head with abundant erect to suberect hairs; hairs on frons slightly sparser; scapes and lateral margin of mandibles with dense decumbent hairs. Dorsal and lateral faces of promesonotum with long erect hairs and short suberect hairs; propodeum with very sparse hairs. Hairs on waist and gaster like major worker. ***Color.*** Whole body yellowish white.

#### Etymology.

The specific epithet *laeviceps* refers to the smooth and shiny head of the major workers.

#### Biology.

Little known, the type material was collected in the grassland of Hanlin Village, Kaijiang City. The species nests underground and feeds on small invertebrates. Some major workers exhibit a swollen gaster, serving as a storage organ for reserves during foraging.

#### Remarks.

*Carebaralaeviceps* is most similar to *C.lusciosa*, *C.bouvardi* (Santschi, 1913) and *C.rectangulata* Bharti & Kumar, 2013, but can be easily distinguished from these three species by combination of the following features: antenna 10-segemented (9-segmented in *C.lusciosa*, *C.bouvardi*, and *C.rectangulata*); posterior margin of head without a transverse carina in major worker (with a transverse carina in *C.rectangulata*); lateral profile of head in major worker parallel in full face view (subparallel in *C.lusciosa*); katepisternum finely rugose-reticulate in major worker (smooth in *C.lusciosa*, punctured in *C.rectangulata*); ventral face of petiole moderately convex (straight in *C.bouvardi* and *C.rectangulata*); distinctly larger with TL ~ 2.8 mm (*C.lusciosa*: 2 mm, *C.rectangulata*: 2.41 mm, *C.bouvardi*: ~ 2.4 mm).

##### ﻿Taxonomic checklist of *Carebara* species in China

A checklist of all known *Carebara* species in China is presented here based on Xu (2003), Zhou et al. (2006), and Terayama et al. (2012). The changes in taxonomic status, diagnostic features and distribution data of each species are provided. The checklist is arranged alphabetically.

***C.acutispina* (Xu, 2003)**

*Oligomyrmexacutispinus* Xu, 2003: 315, figs 16–19 (s.w.) China (Yunnan). Indomalaya.

*Carebaraacutispina* (Xu, 2003). Combination in *Carebara*: Guénard and Dunn 2012: 41.

**Geographic distribution.** China (type locality. Sichuan, Yunnan).

**References.**Xu (2003); Fontanilla et al. (2019); He et al. (2020); Liu et al. (2020); Hosoishi et al. (2022).

***C.affinis* (Jerdon, 1851)**

*Oecodomaaffinis* Jerdon, 1851: 110 (s.w.) India. Indomalaya.

*Pheidoleaffinis* (Jerdon, 1851). Combination in *Pheidole*: Smith 1858: 174.

*Pheidologetonaffnis* (Jerdon, 1851). Combination in *Pheidologeton*: Roger 1863: 30.

*Carebaraaffinis* (Jerdon, 1851). Combination in *Carebara*: Fischer et al. 2014: 71.

**Geographic distribution.** Widespread in Australasia and Indomalaya region: Bangladesh, Borneo, China (Guangdong, Guangxi, Hainan, Hong Kong, Taiwan, Xizang, Yunnan), India (type locality), Indonesia, Laos, Malaysia, Myanmar, Nicobar Island, Philippines, Sri Lanka, Thailand, Australia, Papua New Guinea.

**References.**Zhou and Zheng (1997); Zhou (2001); Lin and Wu (2003); Zhou et al. (2006); Terayama (2009); Guénard and Dunn (2012); Liu et al. (2020).

***C.altinodus* (Xu, 2003)**

*Oligomyrmexaltinodus* Xu, 2003: 312, figs 5–8 (s.w.) China (Yunnan). Indomalaya.

*Carebaraaltinodus* (Xu, 2003). Combination in *Carebara*: Guénard and Dunn 2012: 41.

**Geographic distribution.** China (type locality. Hainan, Jiangxi, Xizang, Yunnan).

**References.**Xu (2003); Chen et al. (2011); Guénard and Dunn (2012); Liu (2012); Song et al. (2013); Liu et al. (2016); Lu and Chen (2016); Liu et al. (2017); Fontanilla et al. (2019); He et al. (2020); Lee et al. (2020); Zhang et al. (2022).

***C.amia* (Forel, 1913)**

*Solenopsisamia* Forel, 1913: 191 (q.) China (Taiwan). Indomalaya.

*Aneleusamia* (Forel, 1913). Combination in *Aneleus*: Emery 1923: 60.

*Oligomyrmexamia* (Forel, 1913). Combination in *Oligomyrmex*: Ettershank 1966: 123.

*Carebaraamia* (Forel, 1913). Combination in *Carebara*: Fernández 2004: 235.

**Geographic distribution.** China (type locality. Taiwan).

**References.**Lin and Wu (2003); Terayama (2009).

**Remarks.** This species only with queen caste described and not similar to any known species.

***C.bihornata* (Xu, 2003)**

*Oligomyrmexbihornatus* Xu, 2003: 317, figs 24–27 (s.w.) China (Yunnan). Indomalaya.

*Carebarabihornata* (Xu, 2003). Combination in *Carebara*: Guénard and Dunn 2012: 41.

**Geographic distribution.** China (type locality. Yunnan).

**References.**Xu (2003); Guénard and Dunn (2012); Liu et al. (2020).

***C.capreola* (Wheeler, 1927)**

Oligomyrmex (Hendecatella) capreolus Wheeler, 1927: 93, fig. 5 (s.w.m.) Vietnam. Indomalaya.

*Carebaracapreola* (Wheeler, 1927). Combination in *Carebara*: Fernández 2004: 235.

**Geographic distribution.** China (Guangdong, Macao), Vietnam (type locality).

**References.**Xu (2003); Guénard and Dunn (2012).

***C.capreolalaeviceps* (Wheeler, 1928)**

Oligomyrmex (Hendecatella) capreolus subsp. laeviceps Wheeler, 1928: 24 (s.) China (Macao).

*Carebaracapreolalaeviceps* (Wheeler, 1928). Combination in *Carebara*: Guénard and Dunn 2012: 41.

**Geographic distribution.** China (type locality. Guangdong, Macao).

**References.**Wheeler (1930); Guénard and Dunn (2012).

***C.castanea* Smith, 1858**

*Carebaracastanea* Smith, 1858: 178 (q.) China (Hong Kong). Indomalaya.

**Geographic distribution.** China (type locality. Hong Kong), Laos, Thailand.

**References.**Xu (1999); Guénard and Dunn (2012).

***C.curvispina* (Xu, 2003)**

*Oligomyrmexcurvispinus* Xu, 2003: 313, figs 9–12 (s.w.) China (Yunnan). Indomalaya.

*Carebaracurvispina* (Xu, 2003). Combination in *Carebara*: Guénard and Dunn 2012: 41.

**Geographic distribution.** China (type locality. Yunnan).

**References.**Xu (2003); Guénard and Dunn (2012).

***C.diversa* (Jerdon, 1851)**

*Oecodomadiversa* Jerdon, 1851: 109 (s.w.) India (Kerala). Indomalaya.

*Pheidolediversa* (Jerdon, 1851). Combination in *Pheidole*: Smith 1858: 174.

*Pheidologetondiversa* (Jerdon, 1851). Combination in *Pheidologeton*: Roger 1863: 30.

*Carebaradiversa* (Jerdon, 1851). Combination in *Carebara*: Fischer et al. 2014: 71.

**Geographic distribution.** Widespread species, mainly in Indomalayan region: Bangladesh, Borneo, Cambodia, China (Fujian, Guangdong, Guangxi, Hainan, Hong Kong, Macao, Taiwan, Yunnan), Guinea, India (type locality), Indonesia, Japan, Laos, Malaysia, Myanmar, Philippines, Singapore, Sri Lanka, Thailand, Vietnam.

**References.**Wu and Wang (1995); Zhou and Zheng (1997); Zhou (2001); Lin and Wu (2003); Zhou et al. (2006); Terayama (2009); Guénard and Dunn (2012).

***C.diversadraco* (Santschi, 1920)**

Pheidologetondiversusst.draco Santschi, 1920: 163 (s.w.q.) Vietnam. Indomalaya.

*Pheidologetondiversusdraco* Santschi, 1920. Subspecies of *Pheidologetondiversus*: Wheeler 1929: 44.

*Carebaradiversadraco* (Santschi, 1920). Combination in *Carebara*: Fischer et al. 2014: 71.

**Geographic distribution.** China (Guangdong, Hainan), Vietnam (type locality).

**References.**Wheeler (1930); Zhou et al. (2006); Guénard and Dunn (2012).

***C.diversalaotina* (Santschi, 1920)**

Pheidologetondiversusvar.laotina Santschi, 1920: 162 (s.w.q.) Laos, Vietnam. Indomalaya.

*Pheidologetondiversuslaotina* Santschi, 1920. Subspecies of *Pheidologetondiversus*: Wheeler 1930: 68.

*Pheidologetonlaotina* (Santschi, 1920). Status as species: Ettershank 1966: 119 (error).

*Carebaradiversalaotina* (Santschi, 1920). Combination in *Carebara*: Fischer et al. 2014: 71.

**Geographic distribution.** Cambodia, China (Fujian, Guangdong, Hongkong, Macao), Laos (type locality), Vietnam (type locality).

**References.**Wheeler (1930); Zhou et al. (2006); Huang and Zhou (2007); Guénard and Dunn (2012).

***C.hunanensis* (Wu & Wang, 1995)**

*Oligomyrmexhunanensis* Wu & Wang, 1995: 75, figs 90, 93 (s.w.) China (Hunan). Indomalaya.

*Carebarahunanensis* (Wu & Wang, 1995). Combination in *Carebara*: Guénard and Dunn 2012: 41.

**Geographic distribution.** China (type locality. Hong Kong, Hunan).

**References.**Xu (2003); Guénard and Dunn (2012).

***C.jiangxiensis* (Wu & Wang, 1995)**

*Oligomyrmexjiangxiensis* Wu & Wang, 1995: 75, 194, figs 91, 94 (s.w.) China (Jiangxi). Indomalaya.

*Carebarajiangxiensis* (Wu & Wang, 1995). Combination in *Carebara*: Guénard and Dunn 2012: 41.

**Geographic distribution.** China (type locality. Guangdong, Jiangxi, Sichuan, Yunnan, Zhejiang).

**References.**Xu (2003); Zhao et al. (2009); Guénard and Dunn (2012); Staab et al. (2014); Huang et al. (2019); He et al. (2020).

***C.latinoda* (Zhou & Zheng, 1997)**

*Pheidologetonlatinodus* Zhou & Zheng, 1997: 165, figs 4–6 (s.w.) China (Guangxi). Indomalaya.

*Carebaralatinoda* (Zhou & Zheng, 1997). Combination in *Carebara*: Fischer et al. 2014: 72.

**Geographic distribution.** China (type locality. Guangdong, Guangxi).

**References.**Zhou (2001); Zhou et al. (2006); Guénard and Dunn (2012).

***C.lignata* Westwood, 1840**

*Carebaralignata* Westwood, 1840: 86, pl. 2, fig. 6 (q.) Indonesia (Java). Indomalaya.

**Geographic distribution.** Widespread in Indomalaya region: Bangladesh, China (Yunnan), India, Indonesia (type locality), Nepal.

**References.**Xu (1999); Guénard and Dunn (2012); Song et al. (2013); Lu et al. (2017).

***C.lusciosa* (Wheeler, 1928)**

*Oligomyrmexlusciosus* Wheeler, 1928: 22 (s.w.) China (Guangdong). Indomalaya.

*Carebaralusciosa* (Wheeler, 1928). Combination in *Carebara*: Fernández 2004: 235.

**Geographic distribution.** China (type locality. Guangdong).

**References.**Xu (2003); Guénard and Dunn (2012).

***C.melasolena* (Zhou & Zheng, 1997)**

*Pheidologetonmelasolenus* Zhou & Zheng, 1997: 163, figs 1–3 (s.w.) China (Guangxi). Indomalaya.

*Carebaramelasolena* (Zhou & Zheng, 1997). Combination in *Carebara*: Fischer et al. 2014: 72.

**Geographic distribution.** China (type locality. Chongqing, Guangxi, Hainan, Henan, Hong Kong, Hubei, Hunan, Jiangxi, Sichuan, Yunnan, Zhejiang).

**References.**Zhou (2001); Zhang and Zheng (2002); Zhou et al. (2006); Huang and Zhou (2007); Guénard and Dunn (2012); Staab et al. (2014); Liu et al. (2015); Liu et al. (2020).

**Remarks.** The status of this species is somewhat ambiguous, In Zhou and Zheng (1997) and Zhou et al. (2006), this species can be distinguished from *C.vespillo* (Wheeler, 1921) by the following characteristics: the coarse black line present in the median longitudinal groove of the head; postpetiolar node distinctly broader than long; and hairs sparser on the head and body. However, in Chen et al. (2021), *C.vespillo* was recorded with the presence of the black line. Accordingly, some former specimens of *C.vespillo* may have been misidentified as *C.melasolena* due to the presence of the black line. In Zhou and Zheng (1997), the authors pointed the postpetiolar node of *C.melasolena* is 1.5× broader than long, in Zhou et al. (2006) the node is 2× broader than long, maybe this ratio is a also an unstable morphological trait.

Above all, the features and separation of these two species needs further examination of the type specimens, it is possible that *C.melasolena* is a synonym of *C.vespillo*, but here we still list *Carebaramelasolena* as a valid species based on former studies.

***C.nanningensis* (Li & Tang, 1986)**

*Pheidologetonnanningensis* Li & Tang, 1986: 162 (s.w.) China (Guangxi). Indomalaya.

*Carebarananningensis* (Li & Tang, 1986). Combination in *Carebara*: Fischer et al. 2014: 72.

**Geographic distribution.** China (type locality. Guangxi).

**References.**Zhou and Zheng (1997); Zhou et al. (2006); Guénard and Dunn (2012).

***C.obtusidenta* (Xu, 2003)**

*Oligomyrmexobtusidentus* Xu, 2003: 316, figs 20–23 (s.w.) China (Yunnan). Indomalaya.

*Carebaraobtusidenta* (Xu, 2003). Combination in *Carebara*: Guénard and Dunn 2012: 41.

**Geographic distribution.** China (type locality. Hunan, Chongqing, Sichuan, Xizang, Yunnan), India.

**References.**Xu (2003); Huang (2005); Chen et al. (2011); Guénard and Dunn (2012); Liu (2012); Song et al. (2013); Fontanilla et al. (2019); Luo et al. (2019).

***C.oni* (Terayama, 1996)**

*Oligomyrmexoni* Terayama, 1996: 20, figs 38–43 (s.w.) Japan. Palearctic.

*Carebaraoni* (Terayama, 1996). Combination in *Carebara*: Terayama 2009: 151.

**Geographic distribution.** China (Taiwan), Japan (type locality).

**References.**Lin and Wu (2003); Terayama (2009); Guénard and Dunn (2012); Terayama et al. (2012).

***C.pseudolusciosa* (Wu & Wang, 1995)**

*Oligomyrmexpseudolusciosus* Wu & Wang, 1995: 76, 195, figs 92, 95 (s.w.q.) China (Hubei, Anhui). Indomalaya.

*Carebarapseudolusciosa* (Wu & Wang, 1995). Combination in *Carebara*: Guénard and Dunn 2012: 41.

**Geographic distribution.** China (type locality. Anhui, Guangxi, Henan, Hubei).

**References.**Xu (2003); Guo (2006); Guénard and Dunn (2012); Lu (2013); Guo et al. (2015).

***C.polyphemus* (Wheeler, 1928)**

*Oligomyrmexpolyphemus* Wheeler, 1928: 21 (s.) China (Guangdong). Indomalaya.

*Carebarapolyphemus* (Wheeler, 1928). Combination in *Carebara*: Fernández 2004: 235.

**Geographic distribution.** China (type locality. Guangdong, Yunnan).

**References.**Xu (2003); Zhao et al. (2009); Guénard and Dunn (2012).

***C.qianliyan*** Terayama, 2009

*Carebaraqianliyan* Terayama, 2009: 152, figs 230, 231 (s.w.) China (Taiwan). Indomalaya.

**Geographic distribution.** China (type locality. Taiwan).

**References.**Terayama (2009); Guénard and Dunn (2012); Terayama et al. (2012).

***C.rectidorsa*** (Xu, 2003)

*Oligomyrmexrectidorsus* Xu, 2003: 319, figs 32–35 (s.w.) China (Yunnan). Palearctic.

*Carebararectidorsa* (Xu, 2003). Combination in *Carebara*: Guénard and Dunn 2012: 41.

**Geographic distribution.** China (type locality. Chongqing, Hainan, Henan, Hubei, Hunan, Sichuan, Xizang, Yunnan), India.

**References.**Xu (2003); Huang (2005); Guo (2006); Guénard and Dunn (2012); Guo et al. (2015); Fontanilla et al. (2019); Huang et al. (2019); Luo et al. (2019); He et al. (2020); Lee et al. (2020).

***C.reticapita* (Xu, 2003)**

*Oligomyrmexreticapitus* Xu, 2003: 319, figs 38–41 (s.w.) China (Yunnan). Palearctic.

*Carebarareticapita* (Xu, 2003). Combination in *Carebara*: Guénard and Dunn 2012: 41.

**Geographic distribution.** China (type locality. Guangxi, Hainan, Sichuan, Xizang, Yunnan).

**References.**Xu (2003); Chen et al. (2011); Guénard and Dunn (2012); Liu (2012); Chen et al. (2013); Song et al. (2013); Cheng et al. (2015); Liu et al. (2016); Liu et al. (2017); Fontanilla et al. (2019); He et al. (2020); Lee et al. (2020).

***C.sakamotoi* Terayama et al., 2012**

*Carebarasakamotoi* Terayama et al., 2012: 2, figs 4–7 (s.w.) China (Taiwan). Indomalaya.

**Geographic distribution.** China (type locality. Taiwan).

**References.** Terayama et al. (2012).

***C.sauteri* (Forel, 1912)**

*Oligomyrmexsauteri* Forel, 1912: 56 (s.) China (Taiwan, Zhejiang). Indomalaya.

*Carebarasauteri* (Forel, 1912). Combination in *Carebara*: Fernández 2004: 235.

**Geographic distribution.** China (type locality. Taiwan, Zhejiang), Japan.

**References.**Lin and Wu (2003); Xu (2003); Terayama (2009); Guénard and Dunn (2012); Terayama et al. (2012).

**Remarks.** In Wu and Wang’s (1992) study, *C.hunanensis* was mistakenly identified as *C.sauteri* but was later corrected by Terayama (Wu and Wang 1995).

***C.striata* (Xu, 2003)**

*Oligomyrmexstriatus* Xu, 2003: 314, figs 13–15 (s.) China (Yunnan). Palearctic.

*Carebarastriata* (Xu, 2003). Combination in *Carebara*: Fernández 2010: 202.

**Geographic distribution.** China (type locality. Sichuan, Yunnan).

**References.**Xu (2003); Guénard and Dunn (2012); He et al. (2020).

***C.taiponica* (Wheeler, 1928)**

Oligomyrmexsilvestriisubsp.taiponicus Wheeler, 1928: 24 (s.) China (Hong Kong). Palearctic.

*Oligomyrmextaiponicus* Wheeler, 1928. Status as species: Bolton 1995: 300.

*Carebarataiponica* (Wheeler, 1928). Combination in *Carebara*: Fernández 2004: 235.

**Geographic distribution.** China (type locality. Hong Kong, Yunnan), Laos.

**References.**Xu (2003); Guénard and Dunn (2012).

***C.trechideros* (Zhou & Zheng, 1997)**

*Pheidologetontrechideros* Zhou & Zheng, 1997: 167, figs 7–9 (s.w.) China (Guangxi). Indomalaya.

*Carebaratrechideros* (Zhou & Zheng, 1997). Combination in *Carebara*: Fischer et al. 2014: 72.

**Geographic distribution.** China (type locality. Guangdong, Guangxi, Hunan, Jiangxi, Sichuan, Yunnan), Thailand, Vietnam.

**References.**Zhou (2001); Zhou et al. (2006); Huang and Zhou (2007); Li et al. (2009); Zhao et al. 2009); Chen et al. (2012); Guénard and Dunn (2012); Song et al. (2013); Zhang et al. (2014); Fontanilla et al. (2019); Huang et al. (2019); Luo et al (2019).

***C.vespillo* (Wheeler, 1921)**

*Pheidologetonvespillo* Wheeler, 1921: 533 (s.w.) China (Zhejiang). Indomalaya.

*Carebaravespillo* (Wheeler, 1921). Combination in *Carebara*: Fischer et al. 2014: 72.

**Geographic distribution.** China (type locality. Guangxi, Henan, Hong Kong, Hunan, Jiangxi, Shandong, Zhejiang), Vietnam.

**References.**Wu and Wang (1992); Bolton (1995); Wu and Wang (1995); Zhou et al. (2006); Guénard and Dunn (2012); Lu (2013); Zhang et al. (2014); Cheng et al. (2015); Guo et al. (2015).

***C.wheeleri* (Ettershank, 1966)**

*Oligomyrmexwheeleri* Ettershank, 1966: 124. Replacement name for *Oligomyrmexsilvestrii* Wheeler, 1928: 23 (s.w.) China (Hong Kong). Palearctic.

*Carebarawheeleri* (Ettershank, 1966). Combination in *Carebara*: Fernández 2004: 235.

**Geographic distribution.** China (type locality Hong Kong, Yunnan).

**References.**Xu (2003); Guénard and Dunn (2012); Huang et al. (2019).

***C.yamatonis* (Terayama, 1996)**

*Oligomyrmexyamatonis* Terayama, 1996: 23, figs 48–51 (s.w.) Japan. Palearctic.

*Carebarayamatonis* (Terayama, 1996). Combination in *Carebara*: Terayama 2009: 151.

**Geographic distribution.** China (Hubei, Hunan), Japan (type locality).

**References.**Lin and Wu (2003); Huang (2005); Guénard and Dunn (2012).

***C.yanoi* (Forel, 1912)**

*Pheidologetonyanoi* Forel, 1912: 57 (w.q.) China (Taiwan). Indomalaya.

*Carebarayanoi* (Forel, 1912). Combination in *Carebara*: Fischer et al. 2014: 72.

**Geographic distribution.** China (type locality. Taiwan).

**References.**Wheeler (1929); Lin and Wu (2003); Zhou et al. (2006); Terayama (2009).

***C.zengchengensis* (Zhou et al., 2006)**

*Pheidologetonzengchengensis*Zhou et al., 2006: 871, figs 1, 2 (s.w.) China (Guangdong). Indomalaya.

*Carebarazengchengensis* (Zhou et al., 2006). Combination in *Carebara*: Fischer et al. 2014: 72.

**Geographic distribution.** China (type locality. Fujian, Guangdong, Macao).

**References.**Zhou et al. (2006); Zhang and Hou (2009); Zhao et al. (2009).

## ﻿Discussion

**Figure 32.**
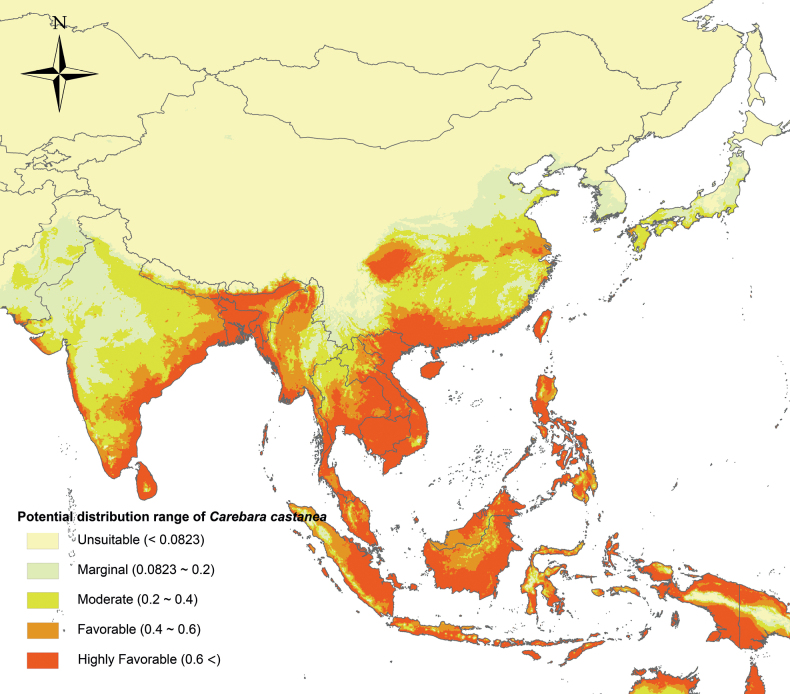
Potential distribution range of *C.castanea* under the current climate.

In this study, a new *Carebara* species, *C.laeviceps* sp. nov. is described and the key and checklist of Chinese *Carebara* species are updated. Chinese *Carebara* species are predominantly small and subterranean, making the collection and identification quite challenging. Previous studies (Liu et al. 2020; Chen et al. 2021) have recorded several undescribed species. As future research advances, certain widely distributed Chinese species, such as *C.melasolena*, may reveal an extensive presence within the Indomalayan region. Furthermore, the Indo-China Peninsula may share certain widespread species with Yunnan and Guangxi provinces, such as *C.castanea* (Fig. 32).

The definition of the *Carebara* species groups is a complex question that requires large-scale research. Current studies, however, are mostly limited to a regional level. Unraveling the phylogenetic relationships among species groups in different faunas also demands a substantial amount of molecular data. Therefore, there is a need for a more comprehensive survey and taxonomic revision of *Carebara* species of the Old World.

A provisional definition of Chinese *Carebara* species groups is provided in this research, and some features of the *concinna-lignata* group have been updated. It is possible that these species might be divided into several distinct groups in future studies; for example, *C.altinodus*, *C.hunanensis*, *C.oni*, and *C.qianliyan* could potentially form a single group due to various shared features, such as a massive mesosoma, the head capsule relatively short (CI > 90), large size (TL > 3.4 mm), and ocelli mostly present. Similarly, there may also be the *acutispina* species group but due to the lack of molecular data and to avoid making polyphyletic groups, we have maintained the classification proposed by Bharti and Kumar (2013).

## Supplementary Material

XML Treatment for Carebara

XML Treatment for Carebara laeviceps

## ﻿Additional information

### Author ORCIDs

Zhi-yu Liu https://orcid.org/0009-0004-1516-6975

Ying Zhong https://orcid.org/0000-0002-6289-4455

Yu-yuan Huang https://orcid.org/0009-0005-3406-4875

Hao Ran https://orcid.org/0000-0001-5462-3615

Fan Song https://orcid.org/0000-0002-2900-4174

### Data availability

All of the data that support the findings of this study are available in the main text.
